# Unmasking the immune microecology of ductal carcinoma in situ with deep learning

**DOI:** 10.1038/s41523-020-00205-5

**Published:** 2021-03-01

**Authors:** Priya Lakshmi Narayanan, Shan E. Ahmed Raza, Allison H. Hall, Jeffrey R. Marks, Lorraine King, Robert B. West, Lucia Hernandez, Naomi Guppy, Mitch Dowsett, Barry Gusterson, Carlo Maley, E. Shelley Hwang, Yinyin Yuan

**Affiliations:** 1 Centre for Evolution and Cancer, Institute of Cancer Research, London, UK; 2 Division of Molecular Pathology, Institute of Cancer Research, London, UK; 3 Department of Pathology, Duke University School of Medicine, Durham, NC USA; 4 Department of Surgery, Duke University School of Medicine, Durham, NC USA; 5 Department of Pathology, Surgical Pathology, Stanford, CA USA; 6 Department of Anatomic Pathology, Hospital Universitario, 12 de Octubre, Madrid, Spain; 7 Breast Cancer Now Histopathology Core, Institute of Cancer Research, London, UK; 8 UCL Advanced Diagnostics, University College London, London, UK; 9 The Breast Cancer Now Toby Robins Research Centre, Institute of Cancer Research, London, UK; 10 Academic Department of Biochemistry, Royal Marsden Hospital, London, UK; 11 Biodesign Center for Personalized Diagnostics and School of Life Sciences, Arizona State University, Tempe, AZ USA

**Keywords:** Breast cancer, Cancer microenvironment, Cancer imaging

## Abstract

Despite increasing evidence supporting the clinical relevance of tumour infiltrating lymphocytes (TILs) in invasive breast cancer, TIL spatial variability within ductal carcinoma in situ (DCIS) samples and its association with progression are not well understood. To characterise tissue spatial architecture and the microenvironment of DCIS, we designed and validated a new deep learning pipeline, UNMaSk. Following automated detection of individual DCIS ducts using a new method IM-Net, we applied spatial tessellation to create virtual boundaries for each duct. To study local TIL infiltration for each duct, DRDIN was developed for mapping the distribution of TILs. In a dataset comprising grade 2–3 pure DCIS and DCIS adjacent to invasive cancer (adjacent DCIS), we found that pure DCIS cases had more TILs compared to adjacent DCIS. However, the colocalisation of TILs with DCIS ducts was significantly lower in pure DCIS compared to adjacent DCIS, which may suggest a more inflamed tissue ecology local to DCIS ducts in adjacent DCIS cases. Our study demonstrates that technological developments in deep convolutional neural networks and digital pathology can enable an automated morphological and microenvironmental analysis of DCIS, providing a new way to study differential immune ecology for individual ducts and identify new markers of progression.

## Introduction

Ductal carcinoma in situ (DCIS) is a non-obligatory precursor of invasive ductal carcinoma (IDC). It is the most common mammographically detected breast cancer, however, predicting DCIS progression to IDC remains a major clinical challenge^1–3^. A recent study has categorised DCIS evolution to IDC into four models, highlighting its heterogeneity. The evolutionary potential of individual DCIS ductules/ducts may dramatically differ, determined by not only their genetic mutations but also microenvironmental selective pressure^4–6^. Remarkable progress in genetic profiling has advanced our understanding of clonal evolution in DCIS. However, given the complex spatial ductule structure, ecological dynamics between individual DCIS ducts and their surrounding microenvironment are difficult to measure by eye. These ultimately limits our ability to study the influence of the microenvironment on tumour evolution and progression^7^.

DCIS lesions are composed of malignant epithelial cells, which proliferate within the breast terminal duct lobular unit and are surrounded by myoepithelial cells and basement membrane. The architectural pattern of DCIS is highly variable, and it broadly comprises solid, cribriform, papillary and comedo type of DCIS^8^. Such diverse patterns of DCIS present challenges not only to diagnosis^9^ but also to the application of machine learning tools.

To the best of our knowledge, few automated methods based on machine learning have been proposed for evaluation of DCIS on haematoxylin and eosin (H&E) samples. One of the approaches used multiscale superpixels to discriminate epithelial area from the remaining tissue area and further clustered the epithelial regions based on a random polygon model, but had difficulty with comedo DCIS^10^. More recently a new deep learning pipeline was developed to predict the risk of recurrence in pure DCIS patients treated with breast-conserving surgery, where texture features were utilised for classification of image patches into the normal duct, stroma, cancer duct and immune rich patterns in H&E^11^. However, such efforts have not yet been applied in the studies of DCIS to evaluate the spatial variability related to individual DCIS ducts with single-cell spatial resolution, or the relationship of these ducts to the immune microenvironment.

Recent studies support the importance of tumour infiltrating lymphocytes (TILs) in the progression from DCIS to IDC^6^ and risk of local and metastatic recurrences^12^. However, TIL assessment still relies heavily on quantification by pathological scoring^13^, which is labour intensive. In this study, we aimed to provide new insights into the local immune microenvironment surrounding individual DCIS ducts by capturing spatial information quantitatively. To automate the spatial mapping of TIL distribution patterns for individual DCIS ducts in H&E, we designed an integrated computational framework based on deep learning. We hypothesise that the local microecology for individual DCIS within the tissue creates differential selective forces and may ultimately influence its potential for progression to invasive cancers. Our primary aims were: (1) to develop and validate a computational pipeline that accurately detects and segments individual DCIS ducts; (2) to characterise the immune microecology for each DCIS duct using spatial statistics on H&E and IHC for TILs; (3) to test the difference in DCIS microecology between samples with pure DCIS and DCIS samples derived from IDC patients (adjacent DCIS, as a surrogate for poor prognosis DCIS).

## Results

### IM-Net for DCIS detection and segmentation

**Fig. 1:**
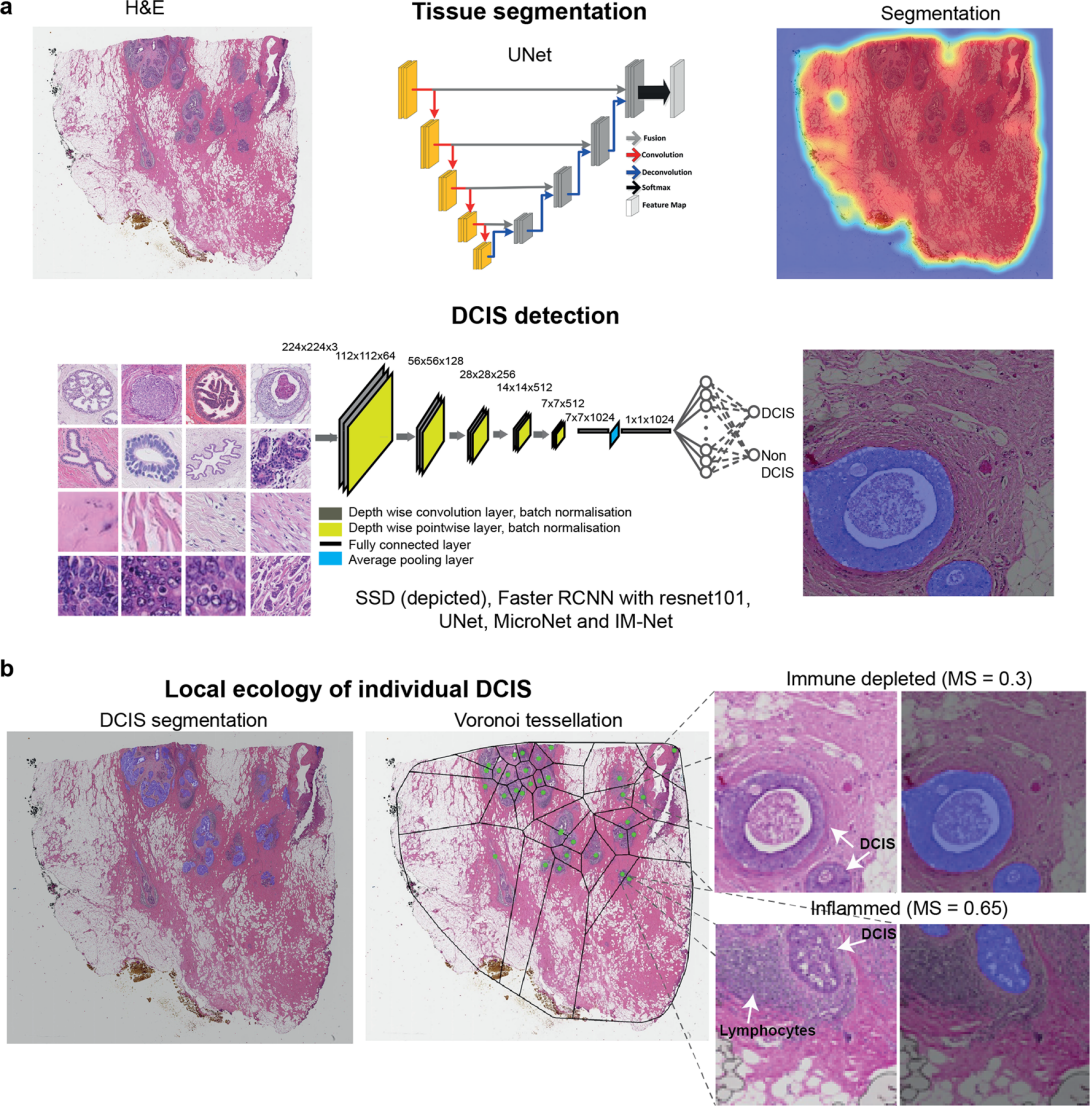
Overview of proposed UNMaSk pipeline for DCIS detection and segmentation. **a** UNet architecture for tissue segmentation and one of the existing deep learning methods, single-shot detector (SSD) architecture, used for DCIS detection. **b** Spatial Voronoi tessellation to examine local tissue ecology for each DCIS duct, based on deep learning results on DCIS segmentation and single-cell classification. Examples shown are immune depleted and immune predominant/inflamed ecology local to individual DCIS ducts and spatial analysis using DCIS immune colocalisation/Morisita Score (MS).

**Fig. 2:**
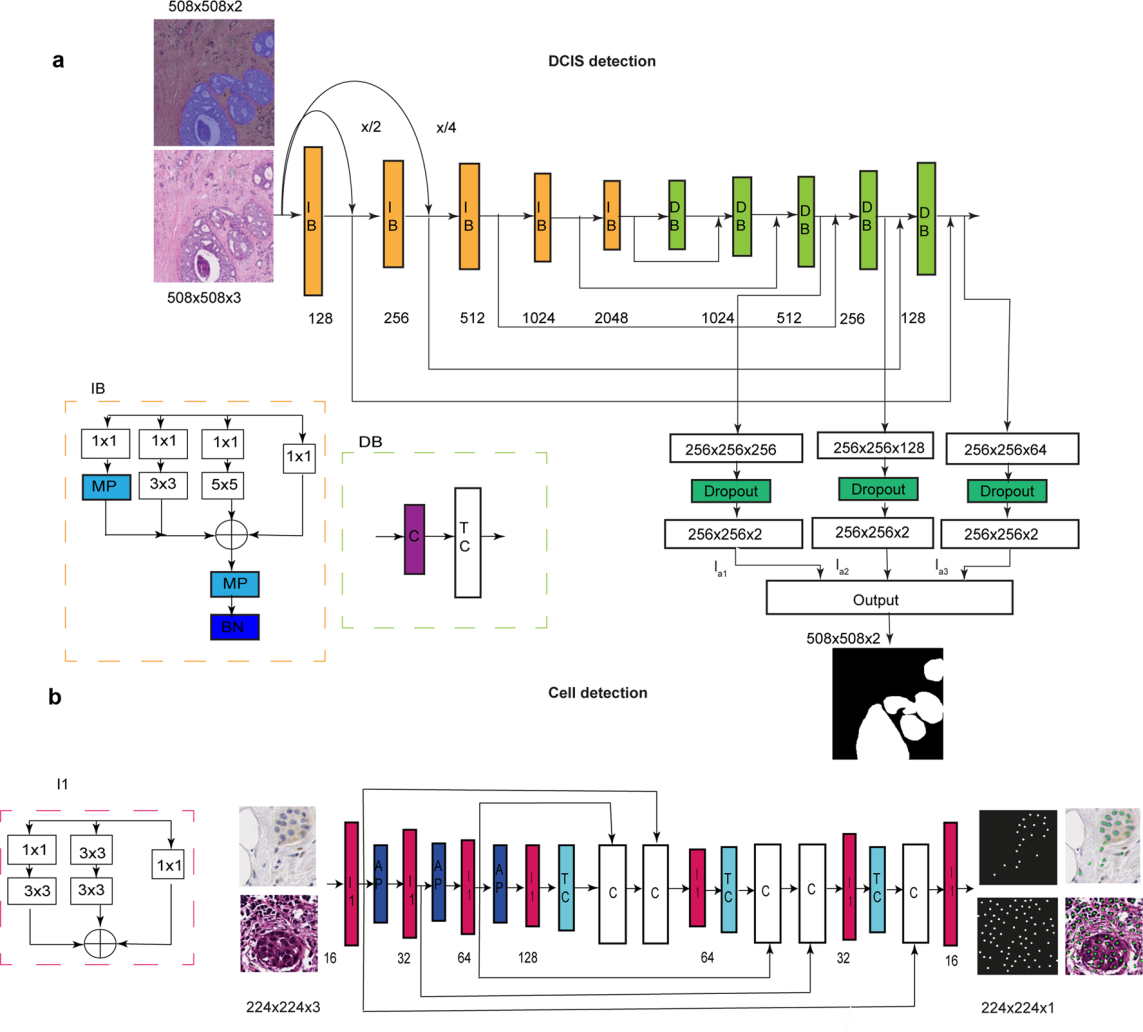
Schematic of IM-Net architecture for detection and segmentation of DCIS and schematic of DRDIN cell detection network. **a** IM-Net architecture. Five inception blocks (IB) in the contracting path and five decoder blocks (DB) in the expanding path used to encode features along with spatial context with multiple inputs applied to the respective first three blocks. Inception blocks with batch normalisation performed on resized images generate feature maps from the convolution blocks (IB_1_, IB_2_ and IB_3_). Resized image by a factor of 2 and 4 are represented as *x*/2 and *x*/4, respectively. Features from the convolution blocks were preserved and passed to the expanding path comprising decoder block with concatenate (C) and transpose convolution block (TC) as the basic units that aid to preserve crucial low-level information for DCIS boundary localisation. **b** DRDIN architecture has a dense cross-connection from the inception blocks (I1) in the encoder and the decoder path. Components of I1 comprises 3 × 3 and 1 × 1 kernel convolutional filters. In the encoder path, average pooling (AP) is used and the decoder path consisted of transpose convolution (TC), concatenate (C) layers.

To automate the identification and segmentation of morphologically heterogeneous DCIS ducts in H&E (Fig. 1), we specifically designed a new deep learning framework, IM-Net (Fig. 2a). IM-Net can: (1) distinguish DCIS by combining high-level spatial context and local features using multiple inputs to the encoders, (2) provide precision in localising DCIS boundary by learning weak boundary features using boundary weight map in the optimisation of features across spatial resolutions, (3) reduce sensitivity to tissue artefacts and local noise by using multiple filters in inception blocks (Fig. 2a). Prior to DCIS detection and segmentation, UNet was first used for tissue segmentation (Fig. 1a). Compared with a threshold-based method, a higher segmentation accuracy for UNet was found (Supplementary Table 1).

To evaluate the performance of the proposed IM-Net in DCIS detection and segmentation, we compared it with five state-of-the-art deep learning methods using images generated across three datasets (Fig. 1a). These include some of the most widely used methods for segmentation or detection: single-shot detector^14^, Resnet 101-based RCNN network^15^, UNet^16^, MicroNet^17^ and the proposed IM-Net. Training, validation and testing samples were split on a patient-level, consisting of 340, 522 and 949 annotations on 40, 20 and 18 H&E whole-section images, respectively (Table 1). Three experiments were conducted to test: (1) accuracy of DCIS detection as a binary segmentation problem; (2) accuracy of DCIS segmentation by quantifying the overlap between pathologists’ delineation and automated segmentation; (3) validation using immunohistochemistry.

**Table 1:** Breakdown of training and validation tiles used in annotations of DCIS.

Category	WSI samples					Number of tiles		Total number of pathologist annotations on tiles
	Duke		TransATAC		IHC			
Training	20		20		0	317		340
Validation	10		10		0	310		522
Test	10		0		8	300		949

Independent held out test dataset (Duke)				Biological validation (IHC)				
HE		100		HE			8	
				IHC			8	

For the first experiment, while IM-Net, MicroNet and Faster RCNN yielded similar precision in the testing set, IM-Net achieved the highest F1-score (0.79, Supplementary Table 2), closely followed by MicroNet (0.77). Next, to compare IM-Net and MicroNet, we performed 3-fold cross-validation within the training dataset split at patient-level. Across all folds, IM-Net achieved higher recall (mean ± standard deviation = 0.860 ± 0.006) compared to recall by MicroNet (0.823 ± 0.036, Supplementary Table 3). Finally, to challenge these two methods, we trained them on TransATAC that had sufficient positive and negative examples, and testing was performed on the DUKE dataset. In this context, IM-Net achieved an F1-score of 0.8 compared to 0.754 for MicroNet (Supplementary Table 4).

**Fig. 3:**
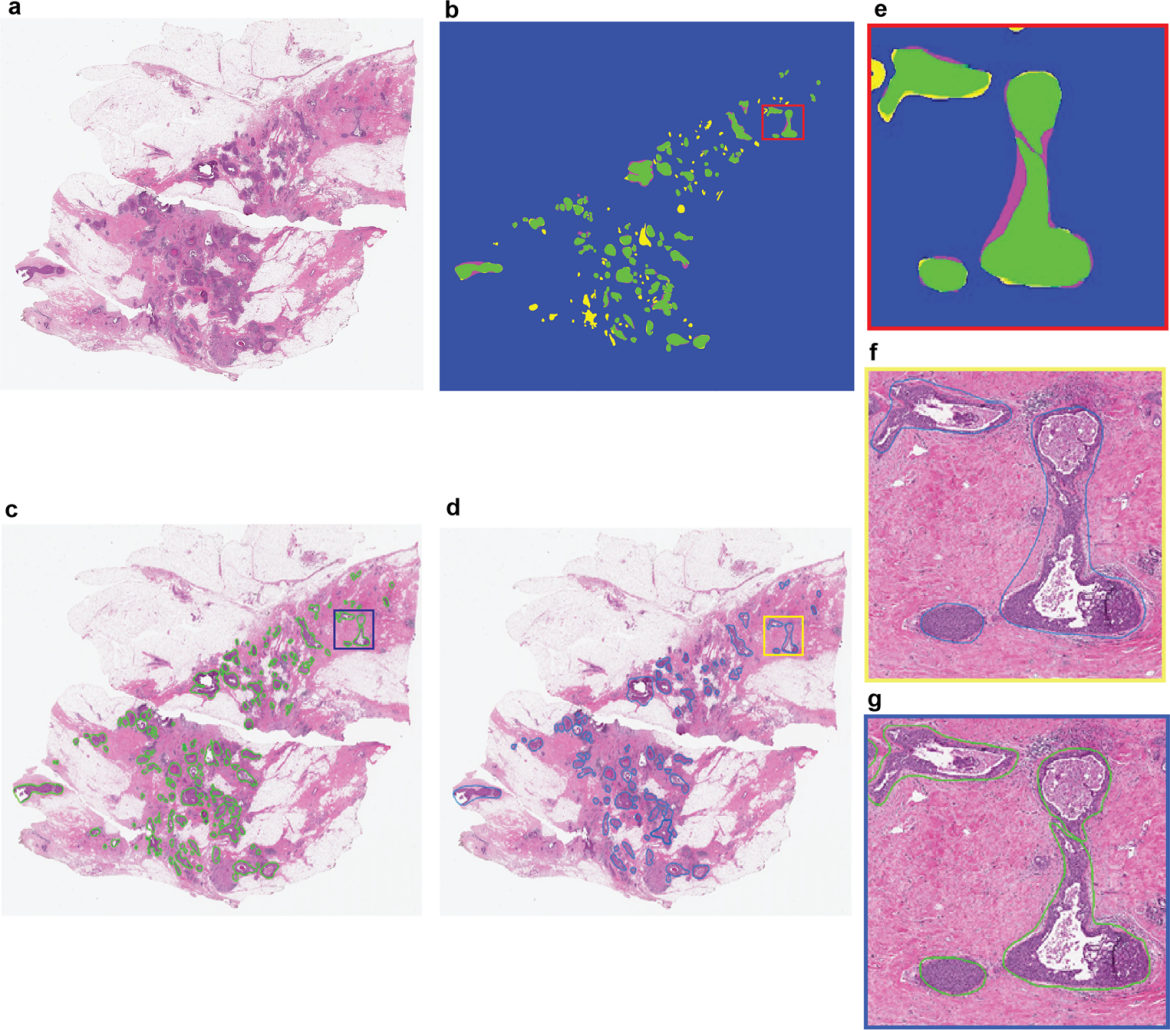
Representative H&E image with DCIS segmentation output from IM-Net. **a** H&E image. **b** Spatial overlap between the pathologist annotation and DCIS detection using IM-Net. Visual representation of DICE overlap image map in terms of estimated DICE coefficient. True positive (TP) in green represent the expert annotations overlap with the segmented region of DCIS; false-negative (FN) in magenta represent DCIS regions in annotation and not in DCIS segmentation. False-positive (FP) in yellow represent pixels falsely segmented as DCIS and not in expert annotation. True negative (TN) represented in blue are pixels were correctly detected as background in both expert and DCIS detection. Inspection of the false-positive regions indicated that some of these were DCIS but contained tissue artefacts or tears, which prevented pathologist from annotating them. **c** DCIS segmentation based on IM-Net. **d** Pathologist annotation of DCIS on H&E image. **e** Region of interest depicting the DICE overlap image map. **f**, **g** Region of interest depicted from pathologist and IM-Net approach, respectively.

Secondly, the overlap measure between pathologists’ annotated DCIS and automated segmentation results indicated that IM-Net followed by MicroNet achieved the best performance among all five methods (0.83 ± 0.30 for IM-Net, 0.82 ± 0.50 for MicroNet, Fig. 3 and Supplementary Table 5). Spatial overlap estimated by DICE for IM-Net was 0.83, where DICE of 0.6 and above is typically considered to indicate a good agreement between ground truth and prediction^18^. Comparison of IM-Net and MicroNet based on spatial overlap suggested that IM-Net can detect the highest proportion of DCIS annotated by pathologists with potentially low false-positive rates. This was supported by a further breakdown of the false-positive and true positive rates (Supplementary Table 5).

**Fig. 4:**
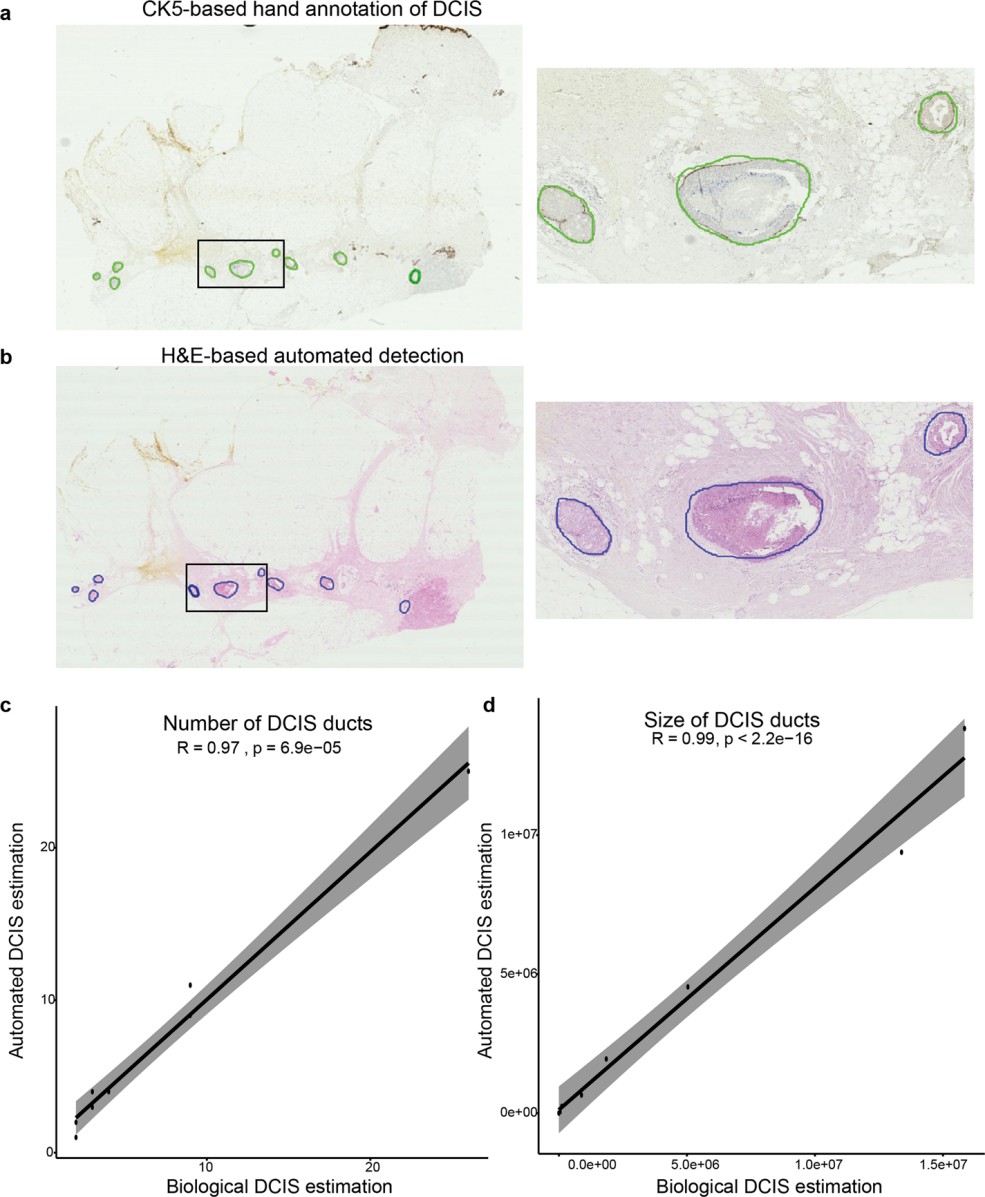
Biological validation of automated DCIS detection using CK5 immunohistochemistry. **a** An example showing CK5 IHC image where DCIS regions were annotated by hand following the CK5 expression pattern, indicated by green contour. **b** Segmented H&E image with DCIS regions marked in blue contour by IM-Net for the same sample. **c** Quantitative assessment of the IHC-H&E correlation between H&E-based automated DCIS detection result and hand annotations on IHC using an estimated number of DCIS. **d** Quantitative assessment of the IHC-H&E correlation between H&E-based automated DCIS detection result and hand annotations on IHC using an estimated area of DCIS.

Finally, further examination of the false-positives of DCIS detected by IM-Net indicated that a number of these were due to tissue artefacts or tears occurring within or near DCIS, which prevented pathologists from annotating them. We, therefore, performed immunohistochemistry experiments to base our validation on biological marker-based expression (see ‘Methods’ section). This biological validation demonstrated a high correlation between IM-Net and MicroNet segmentation of DCIS with hand annotation based on cells positive for CK5 expression lining the basal membrane (cor=0.99 vs cor=0.98–0.94 for other networks, Supplementary Table 6). Detailed performance measures indicate that although MicroNet achieved similar scores as IM-Net in terms of detecting the correct number of DCIS and estimated area, IM-Net was able to more precisely segment the ducts as suggested by the lower mean squared error and higher *R*-square estimate (IM-Net MSE = 0.17, *R*^2^ = 0.65; MicroNet MSE = 0.25, *R*^2^ = 0.59, Supplementary Table 6). Visual inspection confirmed the quantitative data, showing a good agreement between automated detection on the H&E and CK5-based annotations on serial IHC, irrespective of different growth patterns (Fig. 4).

### TILs distribution mapped by deep learning

**Fig. 5:**
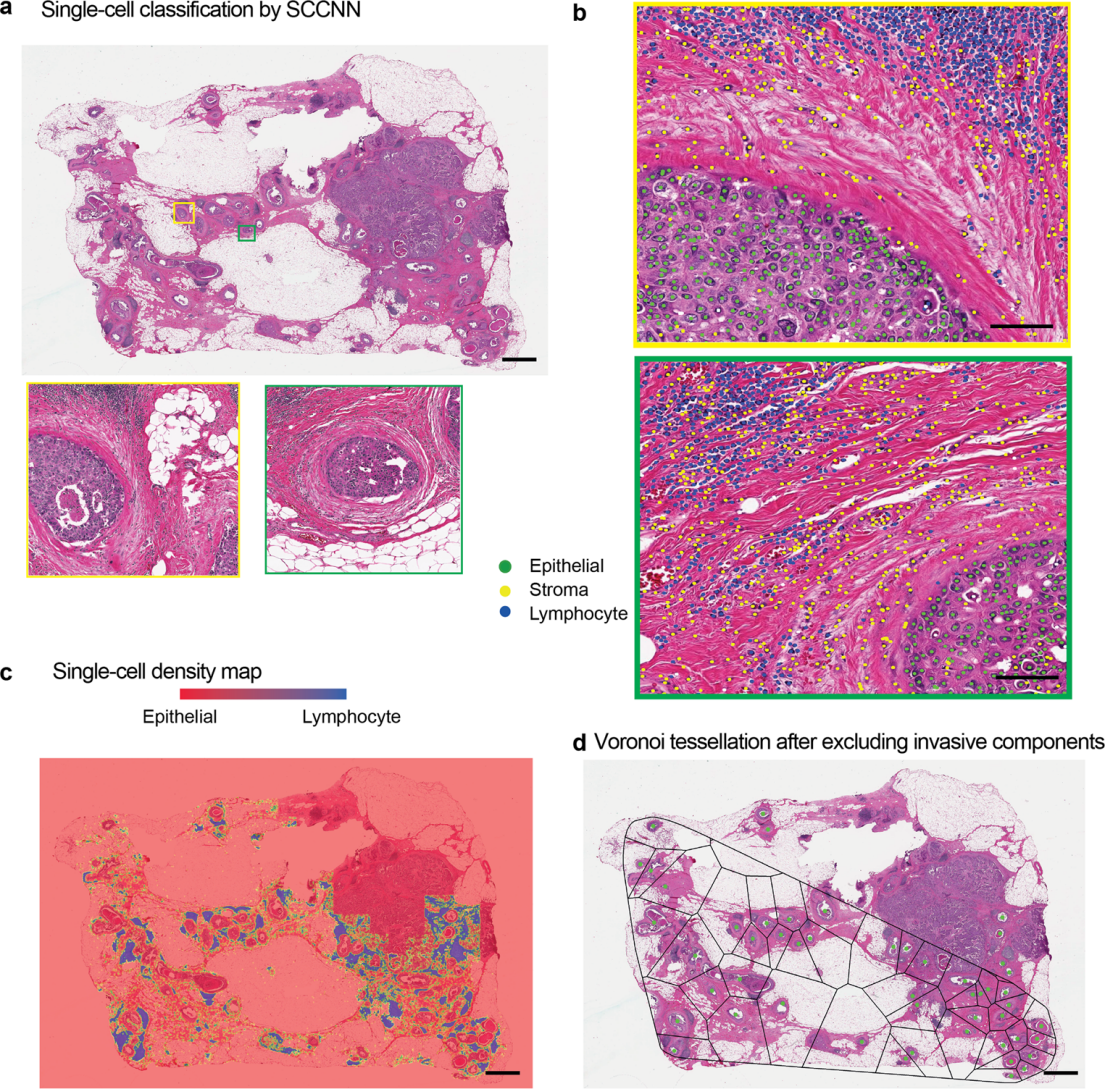
UNMaSk results with single-cell classification and Voronoi tessellation. **a** A representative example of an adjacent DCIS case illustrating single-cell classification results in two DCIS regions. Scale bar represents 100 µm. **b** High-resolution images of areas within the two DCIS regions, showing single-cell classification using unified segmentation and classification pipeline based on DRDIN and SCCNN, classifying cells into the epithelial cell (green), stromal cell (yellow) and lymphocyte (blue). Scale bar represents 50 µm. **c** Heatmap showing lymphocyte cell density based on single-cell classification results. **d** Voronoi tessellation using the centres of DCIS ducts as seeds, performed over tissue region excluding epithelial cells identified by single-cell classification that was not DCIS. Because of the mathematical principles underlying Voronoi tessellations, lymphocytes within a polygon will be closer to its seed than any other seeds. This means that each lymphocyte can now be assigned to its closest DCIS duct within the tessellation space, thereby quantifying lymphocyte abundance for each DCIS duct locally. Note that because convex polygon was used, some of the DCIS ducts closer to the invasive region were omitted from the analysis. Scale bar represents 100 µm.

To spatially map immune and stromal cells that form the immediate microenvironment of DCIS, we tailored a deep learning approach with two steps. First, using Distance Regularised Dense Inception Net (DRDIN), single cells from H&E slides were detected (Fig. 2b). Second, the detected cells were classified based on a neighbourhood ensemble approach implemented in the SCCNN method. Single-cell annotations by experts in the Duke cohort were used for training, validation and testing, comprising a total of 11,412 cells from 12 WSI for training, 5207 cells from three WSI for validation and 5304 annotations from five WSIs for testing. For H&E cell detection, DRDIN achieved an accuracy, recall and F1-score of 0.8065, 0.911 and 0.856, respectively. For H&E cell classification, SCCNN achieved a balanced accuracy of 89.3% in the training cohort and 87.1% using 8302 cells in the TransATAC cohort. As a result, all cells in the H&E images classified into epithelial, stromal and lymphocyte cells (Fig. 5a–c). TILs from H&E image referred to as lymphocyte% was quantified as a percentage of all cells that were lymphocytes for each image.

Using the same network, DRDIN, we collected 4275 cell annotations for training and 754 cells (~15% split) for validation to train a model for immune markers of IHC samples. In addition, we used 8,165 cell annotations for testing, achieving an accuracy, recall and F1-score of 0.8736, 0.936 and 0.9037, respectively. Using this model, all positive cells in the CD4, CD8, FOXP3-stained IHC dataset were identified, and the remaining cells were classified as negative lymphocytes, epithelial cells and stromal cells. We observed a good concordance between H&E-based lymphocyte estimate and IHC-based CD4, CD8, FOXP3 estimate (correlation between lymphocyte% from H&E and CD4^+^% in IHC *R* = 0.86 and *p* = 0.024; CD8^+^% *R* = 1 *p* = 0.0004; FOXP3^+^% *R* = 0.79 *p* = 0.048). This integration of H&E and IHC data provided additional data supporting the accuracy of single-cell analysis using our proposed network that is generalisable to different histology staining.

### Increased colocalisation of TILs and adjacent DCIS

**Fig. 6:**
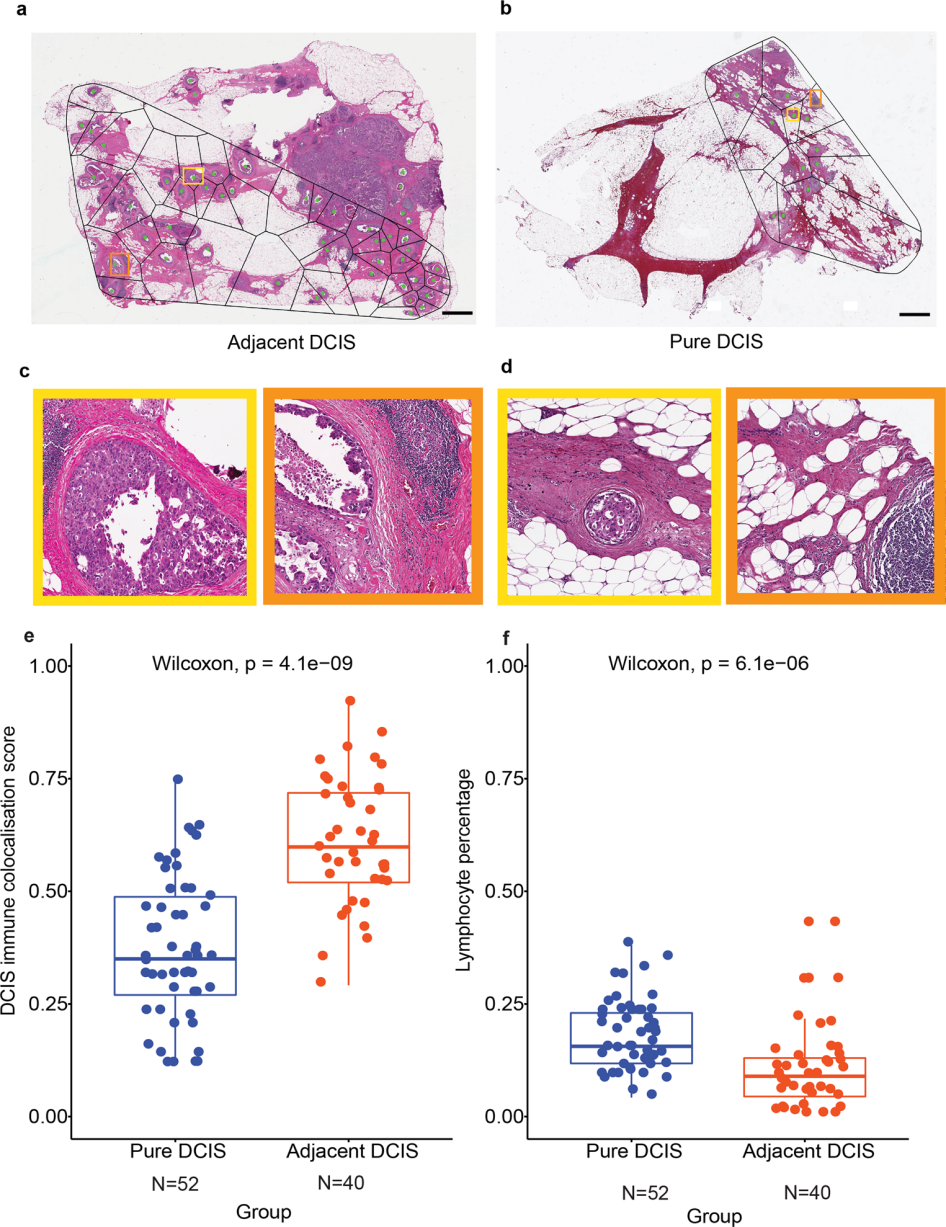
Comparison of TIL distribution pattern local to DCIS ducts in adjacent versus pure DCIS cases. **a** Voronoi tessellation of adjacent DCIS excluding invasive components. **b** Voronoi tessellation of pure DCIS. Scale bar represents 100 µm. **c** Representative DCIS region enclosed within the Voronoi of adjacent DCIS. **d** Representative DCIS region enclosed within the Voronoi of pure DCIS. **e** Boxplots illustrating the difference in DCIS immune colocalisation score calculated using the Morisita index. It was computed by associating individual DCIS duct with the surrounding lymphocytes within the Voronoi region; a high score indicates the spatial colocalisation of lymphocytes and DCIS ducts. Each point corresponds to a WSI image, 52 WSI from *n* = 40 patients in the pure DCIS and 40 WSI from *n* = 25 patients in the adjacent DCIS group. **f** Boxplots illustrating the difference in overall lymphocyte percentage in all cells for WSIs of pure DCIS and adjacent DCIS cases (after exclusion of invasive tumour regions), using only single-cell classifications.

Spatial variability of TILs among DCIS ducts could inform differential ecological features that ultimately dictate invasive potential and fundamental biological underpinning. However, this would require quantitative methods to examine the spatial heterogeneity, instead of focusing on only the abundance of lymphocyte cells. Therefore, to characterise local tumour ecology of individual DCIS, we applied a spatial statistical method, the Morisita index to quantify the colocalisation between TILs and DCIS ducts^19^. Widely applied in ecology, Morisita index is often used to examine spatial overlap or colocalisation. Here it provides a single score for a spatial area: a high score indicates that the two spatial variables, DCIS ducts and TILs, tend to colocalise, whereas a low score suggests TIL exclusion from DCIS or concentration in only a subset of ducts. We applied this method to the Duke dataset to compare pure DCIS with DCIS adjacent to an invasive component as a surrogate for poor prognosis DCIS. The colocalisation pattern of TILs and DCIS ducts in pure DCIS samples were compared with adjacent DCIS in a total of 92 WSIs, representing *n* = 26 pure and *n* = 22 adjacent DCIS cases (Fig. 6a–f). Sample variability of WSI and patients is depicted in the flow diagram (Supplementary Fig. 1). WSI with less than 5 ducts was excluded from computing DCIS immune colocalisation.

A significantly higher number of TILs (measured as a percentage of lymphocytes in all cells after exclusion of invasive tumour regions if any) was found in pure DCIS samples than in adjacent DCIS (*p* = 6.1e−06, Cohen’s *d* = 0.47), however, the DCIS immune colocalisation score indicated that colocalisation of TILs was lower in pure DCIS compared with adjacent DCIS patients with IDC (*p* = 4.1e−09, Cohen’s *d* = 0.61, Fig. 6e, f). The difference in DCIS immune colocalisation score remained significant when the DCIS immune colocalisation score was averaged per patient (*p* = 2.7e−06, Supplementary Fig. 2a), randomly sampled from cases with more than 1 slide available (*p* < 0.05 for 100 of 100 random samplings), or randomly sampled from pure cases to match the number of adjacent cases (Supplementary Fig. 2b). This suggests that although there were more TILs in the pure DCIS samples, these TILs did not colocalise well with DCIS ducts. In contrast, TILs sampled from tissues adjacent to IDCs, although fewer in comparison, tend to localise with DCIS, possibly due to heightened inflammatory response. Representative examples with the positioning of lymphoid aggregates in pure DCIS and adjacent DCIS samples are shown in Fig. 6c, d. There was no correlation between TILs abundance and the DCIS immune colocalisation score within each DCIS group (*p* = 0.082 for pure DCIS and *p* = 0.21 for adjacent DCIS), thus the spatial pattern was not dependent on TIL abundance. These quantitative data and empirical observations support the different types of lymphocyte/epithelial interactions in these two types of DCIS, and further highlights the importance of examining spatial heterogeneity beyond cellular abundance alone.

Furthermore, we tested the dependency of the DCIS immune colocalisation score on clinical parameters of these patients including ER, PR, HER2, age and grade (Table 2). Firstly, the DCIS immune colocalisation score continued to differentiate two DCIS types within ER-positive and ER-negative patients (Supplementary Fig. 3). Secondly, in a multivariate model predicting DCIS types with the DCIS immune colocalisation score, TILs abundance and clinical parameters, the DCIS immune colocalisation score remained a significant discriminator of pure, versus adjacent DCIS (*p* = 9.08e−03). These data suggest that the DCIS immune colocalisation score which measures the colocalisation pattern of TILs and DCIS ducts within the tissue may have utility in differentiating pure and adjacent DCIS, independent of known clinical parameters.

**Table 2:** Demographics of patients belonging to DUKE dataset comprising pure DCIS and adjacent DCIS.

Features	Pure DCIS	Adjacent DCIS
*Age (mean* *±* *std)*	56.19 ± 11.47	61.26 ± 7.40
>60	18	14
<60	22	11
*DCIS grade*		
2	17	7
3	23	17
2–3		1
*ER status*		
Positive	27	16
Negative	7	9
Not processed (NP)	6	0
*PR status*		
Positive	27	14
Negative	7	11
Not processed	6	0
*HER2 status*		
Positive	NA	5
Negative	NA	20

### TIL phenotypes in DCIS and invasive region

**Fig. 7:**
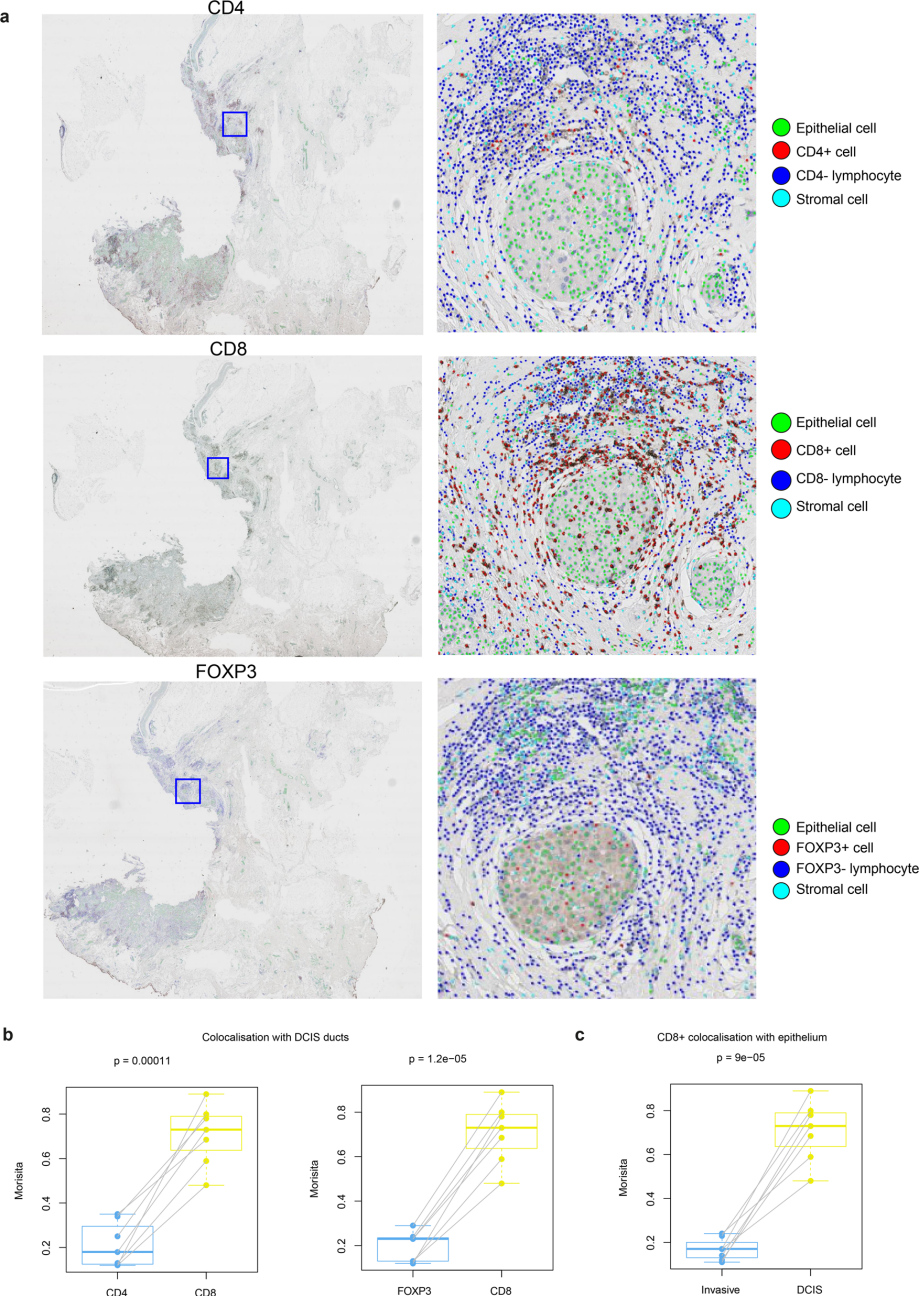
Spatial colocalisation patterns of TIL subset in adjacent DCIS samples in the IHC dataset. **a** Low power CD4, CD8, FOXP3 IHC images of a sample and high-resolution images of a region of DCIS showing single-cell detection and classification using DRDIN and SCCNN. **b** Boxplots showing differences in DCIS immune colocalisation score between CD8^+^, CD4^+^ and FOXP3^+^ cells using paired tests. **c** Boxplots showing the difference in CD8^+^ cell colocalisation between DCIS and invasive epithelium indicating differential immune response within the same sample in different compartments. DCIS immune colocalisation score is referred to as Morisita to conserve space in the representation.

Our data suggest that within the DCIS compartment in adjacent samples, lymphocytes decrease in amount but localise well with DCIS ducts. Increasing evidence supports the importance of T cells in DCIS progression^20,21^. To test whether the colocalisation pattern in adjacent DCIS differs according to T cell subsets, we evaluated the quantitative number, ratio and DCIS immune colocalisation using CD4, CD8 and FOXP3 cells in the IHC dataset of adjacent tumours (Fig. 7a). Following automated identification of cells, we separated the invasive compartment from DCIS compartment as described above, and computed cell percentage within each compartment, as well as colocalisation score between each immune cell subset and DCIS or invasive cancer cells as described previously^19^.

Within the DCIS compartment, DCIS colocalisation score of CD8^+^ cells was significantly higher than CD4^+^ and FOXP3^+^ cells, suggesting that CD8^+^ cells colocalise better with DCIS ducts and may reflect a state of immune activation (Fig. 7b). However, despite having a similar amount in both compartments (*p* > 0.05), CD8^+^ cells colocalised significantly less with invasive cells compared with DCIS (Fig. 7c). In comparison, there was no difference in the colocalisation pattern between compartments for CD4^+^ or FOXP3^+^ cells (Supplementary Fig. 4b) after multiple testing correction (*p* > 0.05). The invasive compartment could be further characterised as having a higher amount of CD4^+^ cells and the ratio of CD4/CD8 than DCIS compartment (Supplementary Fig. 4a, c). These data indicate the immunosuppressive function of invasive cancer cells specifically for CD8^+^ cell infiltration, despite increased immune recognition and localisation of CD8^+^ cells with DCIS epithelial cells.

## Discussion

There is high variability in clinical outcomes and propensity for invasion among DCIS cases. Development of reliable markers, which can identify DCIS patients who are likely to follow a benign course from those who would benefit from therapy are currently an unmet requirement for clinical care.

To provide advanced tools for identifying interactions between individual DCIS and its local immune microenvironment, we developed a multi-stage deep learning framework, UNMaSk that integrates tissue segmentation, DCIS segmentation, single-cell detection, classification and spatial analysis in routine H&E histology images. The framework was developed for the study of tissue microecology by spatially measuring the DCIS and immune colocalisation. As such, our approach can be used to deliver a DCIS immune colocalisation score that integrates morphological context of DCIS and high-resolution single-cell-based classification with intrinsically captured heterogeneous DCIS microenvironment. This type of integrative approaches was not previously possible due to the lack of computational utility, and we bridged this gap in this study.

Routine clinical H&E stained images are known to be highly noisy with staining variability and preprocessing artefacts. Frequently occurring problems in WSI are fixation artefacts, coverslip artefacts and pen marking. Our framework incorporates problem-specific design using tissue segmentation based on UNet to alleviate effects due to artefacts. Training and evaluation were performed on datasets that were processed, stained and scanned in independent laboratories using different digital slide scanners, to reduce overfitting and improve generalisability across scanners and datasets in an unbiased and systematic manner.

Additionally, we showed that the proposed network (IM-Net) for DCIS segmentation was able to capture image semantics across granularities with the usage of inception block and the multiple resized input images fed to each convolution block in the contracting path. The tailored inception block usage in the contracting path and its effective use of the weighted loss function introduced for the first three convolution and transpose convolution blocks preserved features of DCIS. Despite the major challenges in analysing diverse growth patterns across images, the proposed IM-Net has improved DICE and reduced false-positive rate compared to the other networks (Supplementary Table 5). The context features for large and small DCIS were captured from multiple resized images and IM-Net was found to be agnostic to the size and morphology of DCIS. Furthermore, IM-Net could capture weak boundaries of the ducts containing necrosis without over-segmentation of DCIS regions (Supplementary Fig. 5).

Without an automated framework like UNMaSk, it would be impossible to derive precise ecological parameters such as lymphocytic colocalisation with high spatial resolution. Spatial mapping of individual DCIS ducts combined with single-cell classification based on deep learning enabled characterisation of DCIS tissue habitat at micro-scale, i.e. microecology. In the Duke cohort, there is a lower abundance of TILs but higher colocalisation of TILs and DCIS ducts in DCIS adjacent to invasive cancer compared to pure DCIS cases. Increased number of leukocytes is often associated with high-grade DCIS, Her2 status and IDC^6^. Our data showing lower TIL abundance in adjacent DCIS compared with pure DCIS may be attributed to the fact that pure DCIS samples in our cohort were intermediate to high-grade (47% grade 2 and 53% grade 3), and that invasive tumour regions were excluded in the calculation for the adjacent cases. Nevertheless, our observation of differential colocalisation spatial pattern is new. Although high spatial variability of TIL distribution in DCIS has been reported, it has rarely been quantitatively measured. Our data on an increased level of spatial colocalisation of TILs to individual DCIS ducts in adjacent DCIS suggest a potentially different spatial configuration of the immune microenvironment in these patients compared with pure DCIS. Based on these data, we speculate that the colocalisation-based spatial immune score may help predict which DCIS is likely to progress to invasive disease.

Indeed, in a small dataset of adjacent DCIS samples, we observed that despite having equal quantity in the DCIS and invasive compartments, CD8^+^ cells colocalised significantly less with invasive epithelial cells compared to DCIS epithelial cells. To this effect, increased DCIS immune colocalisation score in adjacent DCIS perhaps suggests inflamed local microenvironments for DCIS epithelium but escape from cytotoxic cell recognition in the part of invasive cancer. This will require further validation in larger cohorts, as well as in samples with longitudinal follow up. Nevertheless, our data are consistent with a study where dense chronic inflammation surrounding DCIS, defined as circumferential cuffing of the duct by lymphocytes or plasma cells at least three cell layers in thickness, was associated with a high recurrence score of Oncotype DX DCIS^22^. This preliminary evidence supports future studies to validate the use of the DCIS immune colocalisation score in predicting propensity of individual DCIS for invasive progression. This hypothesis is biologically intriguing, and these studies are currently under way in our lab.

Further study limitations include the lack of high-dimensional immunohistochemistry markers to delineate more subtypes of TILs^21^ beyond CD4/CD8/FOXP3, other key cell types such as macrophages, B cells^20^ and cancer-associated fibroblasts in the tumour ecosystem, as well as the use of 2D slides that do not fully represent the 3D ductal structure. However, methods like UNMaSk provide additional usage of 2D, noisy clinical routine samples by deploying tailored artificial intelligence that could likely help transform traditional pathology. Future studies could focus on longitudinal and 3D sampling to gain a better understanding of the spatio-temporal heterogeneity of TIL distribution across ductal structures.

Little is known in the evolution of immune response and immunologic alterations along the continuum from DCIS to IDC. We envisage that our future opportunities will include the generalisation of our deep learning method to other types of histological images such as immunohistochemistry for defining immune cell subsets surrounding DCIS, investigation of the morphological and architecture details within detected DCIS ducts to account for the types of DCIS, integration with genomic aberrations that could drive progression^23–25^, adaptation for other spatial analyses and scaling up in large patient cohort analyses. A potential new utility of UNMaSk is the detection of DCIS as part of a screening programme, which warrants further investigations.

Thus, our study contributes to driving future research towards the use of novel parameters such as DCIS spatial immune score based on automated histology image analysis for the identification of drivers and biomarkers of progression from DCIS to invasive cancers. This could further potentially aid in stratifying risk of progression, and ultimately improve personalised clinical care.

In summary, we presented a new deep learning pipeline, UNMaSk, for the automated detection and segmentation of DCIS ducts. Our comprehensive evaluation experiments on three different cohorts, using expert annotations and biological immunohistochemistry, and comparison with state-of-the-art convolutional networks demonstrated UNMaSk to be agnostic to diverse size and growth patterns of DCIS. Our framework allowed the integration of spatially heterogeneous DCIS to be associated with lymphocyte distribution by considering the topological arrangement of DCIS. This provides a unique opportunity to study inflammatory response reactive to carcinoma at the high spatial resolution, paving the way for quantitative analysis of DCIS ecology, morphology and AI-aided risk stratification for DCIS disease.

## Methods

We utilised three independent breast tumour datasets, which we referred to as the Duke, TransATAC and IHC datasets. These samples were processed, stained and scanned in independent laboratories using different digital slide scanners.

The Duke dataset consists of samples of pure DCIS disease and DCIS with adjacent invasive cancer, the latter serving as an indicator for poor prognosis DCIS based on the fact that progression to invasive cancer has already occurred. The slides used for the study were selected for the presence of DCIS, determined based on morphology on routine H&E staining according to standard pathology criteria. IHC stains for p63 (a myoepithelial marker) was performed as part of the study. Results based on IHC analysis were used to confirm whether patients belong to DCIS and/or invasive carcinoma (adjacent DCIS) on the analysed slides. H&E stained images acquired from subjects with no invasive component are henceforth referred to as pure DCIS and images from patients with an invasive component along with DCIS are henceforth referred to as adjacent DCIS throughout the manuscript. In total, 140 WSIs were obtained from formalin-fixed paraffin-embedded blocks from 65 patients (*n* = 40 pure and *n* = 25 adjacent), ranging from 1 to 3 slides per patient. These were digitised with an automated whole-slide Aperio scanner at a resolution of 0.5 µm/pixel at ×20 magnification. The study was approved by the institutional review board of Duke with a waiver of the requirement to obtain informed consent. Patient demographics and baseline characteristics of the Duke dataset are summarised in Table 2, and a CONSORT diagram is provided in Supplementary Fig. 1.

TransATAC is the translational clinical study of the Arimidex, Tamoxifen, Alone or in Combination (ATAC) trial on postmenopausal patients with ER^+^ breast cancer treated with tamoxifen or anastrozole^26^. Patient demographics information can be found in Supplementary Table 1 in refs. ^26,27^. 30 WSI H&E images representing 30 invasive breast carcinomas in the TransATAC study were selected, which contained adjacent DCIS. The study was approved by the South-East London Research Ethics Committee, and all patients included gave informed consent. Images from TranATAC dataset were digitally scanned using Hamamatsu Nanozoomer scanner at a pixel resolution of 0.45 µm/pixel at ×20 magnification.

A set of 8 samples were inspected by pathologists and selected on the basis that they contained confirmed areas of DCIS and IDC in the same tissue specimen as part of a previous study^23^. Patient demographic information can be found in Table 1 in ref. ^23^. H&E staining and immunohistochemistry with CK5, CD4, CD8 and FOXP3 were performed on consecutive 3-µm-thick sections of paraffin-embedded blocks. Samples from the IHC dataset were anonymized prior to analysis and the study was approved by ethical committees at Hospital Universitario 12 de Octubre, Madrid, with a waiver of the requirement to obtain informed consent. For one of the eight samples, quality of CD4, CD8 and FOXP3 staining was deemed unsuitable and thus removed from the analysis. Resulting slides were digitalised with Hamamatsu Nanozoomer whole-slide scanner at a pixel resolution of 0.45 µm/pixel at ×20 magnification.

### Immunohistochemical staining

Immunohistochemistry was carried out using the Leica Bond III automated immunostaining platform using Leica Bond Polymer Refine Detection (Leica Biosystems, DS9800) according to the manufacturer’s instructions. Peroxidase blocking and haematoxylin counterstaining were performed onboard as per kit. All primary antibodies were applied for 15 min at room temperature. CD4 (Leica Biosystems, mouse 4B12, cat. PA0427) and CD8 (Leica Biosystems, mouse 4B11, cat. PA0183) were used as supplied, FOXP3 (Invitrogen, mouse PCH101, cat 14-4776-82) was used diluted 1:100 in Leica Bond Primary Antibody Diluent (Leica Biosystems, AR9352). Heat-mediated epitope retrieval was carried out onboard as follows: CD4 and CD8 using Leica Bond ER2 (high pH, AR9640) for 20 min at 97 °C and FOXP3 using Leica Bond ER1 (low pH, AR9961) for 30 min at 97 °C. Slides for CK5 (Dako, mouse D5/16 B4, M7237) were stained on the Dako Link48 automated immunostaining platform, and primary antibody detected using the Dako Envision FLEX kit (Agilent, K8023) according to manufacturer’s instructions. Peroxidase block and haematoxylin counterstaining performed onboard as per kit. Primary antibody was diluted 1/25 in Dako FLEX primary antibody diluent (Agilent, K8006) and applied for 60 min at room temperature. Heat-mediated epitope retrieval was performed using the Dako PT LINK module using EnVision FLEX Target Retrieval Solution, High pH for 20 min at 97 °C.

### Manual annotation collection protocol

Ground truth annotations for DCIS ducts were hand marked by an expert pathologist (B.G.) on the WSI acquired from both Aperio and Hamamatsu scanners. We then used customised scripts to export raw annotations from Aperio Imagescope. DCIS often involves multiple ductules within a single lobule. When this occurs multiple ductules are often seen as individual duct-like structures filled with cancer cells and surrounded by a myoepithelial layer and basement membrane but separated by connective tissue^28^. These biological features of DCIS informed our experimental design. Annotations of individual DCIS ducts, together with annotations of single cells (epithelial cells, lymphocytes, stromal cells), were used for training deep learning networks. Subsequently, we split each of these WSI images into tiles of size 2000 × 2000 pixels (1000 µm × 1000 µm). The tiles were subsequently converted into image patches of different sizes to cater for our training networks (detailed for individual methods). Quality control of annotations was performed at the tile level and an expert consensus was obtained before using the respective tiles for training.

### Training and validation of DCIS

**Fig. 8:**
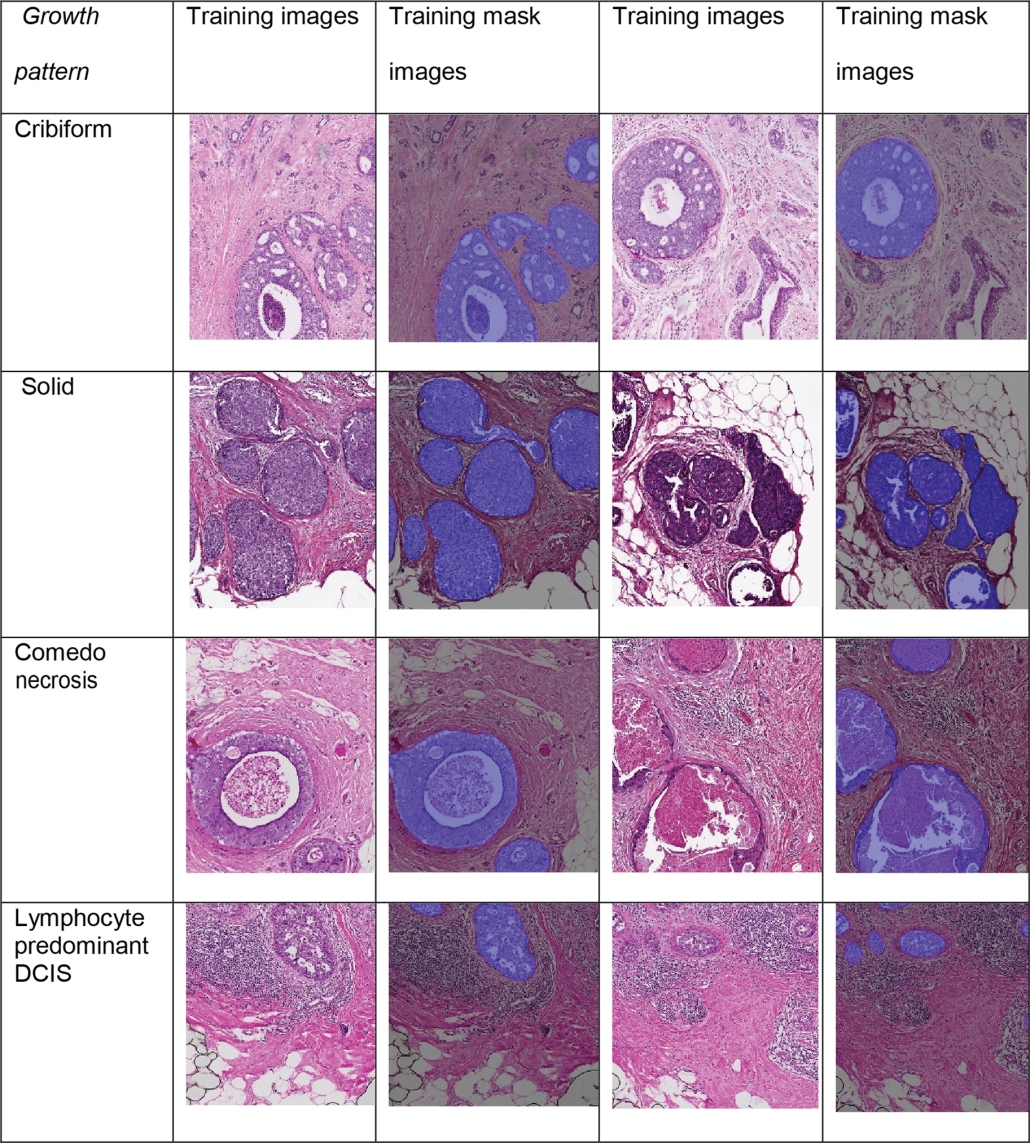
Representative examples of DCIS from the Duke and TransATAC cohorts with different growth patterns.

We randomly selected 20 WSIs from the Duke and 20 WSIs from the TransATAC datasets for training the deep learning methods. The validation dataset consisted of 10 WSIs from Duke and 10 WSIs from TransATAC. 10 WSIs with annotations from Duke were used as the test dataset. Furthermore, 8 WSIs from the IHC dataset were used as an independent test set. Training, validation and test data splitting was performed at the patient-level. Further breakdown of the number of annotated tiles employed in training, validation and testing are tabulated in Table 2. Representative images from these datasets indicating varying growth patterns and DCIS surrounded by different microenvironments are shown in Fig. 8. Training images include both positive and negative examples (Supplementary Fig. 6). The selection of the negative examples was guided by an expert pathologist to include invasive regions, pure stromal, background regions and challenging regions that are visually similar to DCIS such as dense invasive region and lymphocytic clusters. To overcome sample imbalance issue, training images were sampled to generate a balanced number of positive and negative examples.

### UNMaSk framework

The proposed UNMaSk (UNet-IM-Net-SCCNN) framework was designed to integrate multi-stage convolutional networks to: (1) identify tissue and remove background and large artefacts using UNet; (2) detect and segment DCIS regions using a new Inception MicroNet (IM-Net); (3) detect single cells using a new Distance Regularised Dense Inception Network (DRDIN). Cells are then classified using an existing model, Spatially Constrained Convolutional Neural Network (SCCNN); (4) exclude invasive region from DCIS in adjacent samples; (5) perform spatial tessellation to identify immune cells in the vicinity of individual DCIS ducts (Fig. 1).

### UNet for tissue detection

We used generic UNet encoder–decoder architecture^16^ to perform tissue segmentation at ×1.25 resolution of the image based on the openslide library^29^. We fine-tuned the network for tissue segmentation with a patch size of 512 × 512. Fifty training images were selected at random from the datasets, followed by standardised mean normalisation. The experiments used a learning rate of 0.001 for 50 epochs with 20 percent hold out dataset for validation.

### IM-Net for DCIS detection

The proposed IM-Net architecture design includes contracting path, which acts an encoder, and the expanding path, which acts as a decoder of features from the network (Fig. 2a). We used five inception blocks in the contracting path and five decoder blocks in the expanding path where data were encoded along with the spatial context with multiple inputs applied to the first three encoder blocks of IM-Net. To simultaneously gain local features and wider context at the same level, we introduced a custom inception block with batch normalisation performed on resized images to generate feature maps from the convolution blocks (IB_1_, IB_2_ and IB_3_). Each inception block of our IM-Net (Fig. 2a) used the 1×1 convolution to extract features at different scales of the input. The parallel paths in the inception block aid to concatenate all the features from four different scales to feed to individual blocks in the contracting path. Features from the convolution blocks were preserved and passed to the expanding path to preserve crucial low-level information. Low-level information is particularly important for segmentation and by concatenating and upsampling these features at different resolutions, the network learns to preserve weak boundary features. Learning weak boundary features is a specific challenge for DCIS regions with necrosis, thus it is particularly important to address this to achieve consistent segmentation of DCIS.

Input image tiles were of size 2000 × 2000, from which patches of size 508 × 508 were extracted. The rationale behind choosing input patches of size 508 × 508 for IM-Net is to allow the network to capture the representative context of DCIS, achieved from empirical experimentation on patch sizes of 600 × 600, 508 × 508, 512 × 512. Subsequently, input patches were augmented with a random flip, rotation, scaling, gaussian blur, barrel and pincushion distortion. Tiles at the edges of the image were padded with neighbouring patches to ensure all training regions were of size 508 × 508. These were then normalised to zero mean and unit norm and then fed to the network architecture as depicted in Fig. 2a. We used an initial learning rate (*lr*0 = 0.0001) and reduced it through the epochs (*lr* = *lr*0/10^epoch^). The loss function was defined as weighted cross-entropy loss from the main and auxiliary outputs. A post-processing step was used to exclude ducts with less than 10 epithelial cells upon pathologist’s advice.

### Distance regularised dense inception net (DRDIN) for single-cell detection

DRDIN network is comprised of the encoder, decoder and cross-connections that aids in the compilation of the dense UNet with inception blocks (I1) as backend (Fig. 2b). Inspired by the recent improvements based on the inception block that uses parallel paths with the variable convolution filters, we utilised inception V3 as the baseline for each block of the encoder and decoder architecture. Feature reuse by concatenating using dense connection led to better optimisation. Apart from directly adding dense connections in the expansion path, we added inception blocks, which efficiently reduced the number of parameters for optimisation for cell detection.

Network parameters were optimised by minimising the forward Hausdorff distance (HD_f_) as given in Eq. (1), where summation represents the parameter estimated over the batch size (B) of images. ${\mathrm{HD}}_P^j$ and ${\mathrm{HD}}_g^j$ are the Hausdorff distance between the predicted and actual image cell detections. Hausdorff distance between the subsets are taken in the isometric embedding space, which takes into account smaller and the larger distance and are relatively used in the segmentation evaluation metric compared to the Euclidean distance. Compared with a loss function that uses Euclidean distance, the use of Hausdorff distance helped achieve better convergence, because predictions and ground truth cell centroids are not necessarily paired and Hausdorff distance captures the overall geometrical similarity between two sets of points.

1 $${\mathrm{Hausdorff}}\,{\mathrm{distance}}\left( {{\mathrm{HD}}_{{f}}} \right) = 1\frac{1}{{1 + \frac{1}{B}\mathop {\sum }\limits_{j = 1}^B \left| {{\mathrm{HD}}_P^j - {\mathrm{HD}}_g^j} \right|}}.$$

DRDIN was used to detect all types of cells present in H&E and immunohistochemistry images stained for CD8, CD4 and FOXP3 markers. The centroids of the cells were regressed from the probability map of DRDIN where a threshold of 0.8 was empirically chosen to yield centroids of detected cells. We calculated precision, recall and F1-score between the predicted cell centroids and the ground truth cell annotations.

### SCCNN for single-cell classification

Single-cell classification was performed based on detected cell centroid by DRDIN, using SCCNN^30^. The detected cell centroids were then fed to SCCNN classifier. SCCNN classifier network uses neighbouring ensemble prediction combined with the standard softmax for classification. Expert cell annotations were collected and then used for evaluation of classification accuracy. DRDIN detected cells from H&E images were classified into epithelial, stromal and lymphocyte cells. For IHC, we classify the detected cells to epithelial, stain positive lymphocyte, stain negative lymphocyte, and stromal cells.

### Exclusion of invasive regions

Combining the DCIS segmentations from IM-Net and single-cell classifications that identified epithelial cells, we reclassified epithelial cells inside DCIS regions as in situ epithelial cells, and the rest of epithelial cells were reclassified as ‘other’ epithelial cells that could include both invasive and normal ductal cells. Tissue regions containing ‘other’ epithelial cells were deemed invasive compartment, due to the small amount of normal ducts observed in these samples, and removed from polygon analysis (as shown in Fig. 6a).

### Spatial tessellation and the Morisita index

Spatial compartmentalisation was achieved automatically by partitioning DCIS tissue space using Voronoi tessellation, resulting in Voronoi polygons that contain one DCIS duct at the centre. The tessellation is a partition of space according to neighbourhood relations of a given set of points in the space. It has been suggested that Voronoi tessellation mimics the biological patterns present in the histological image and naturally emerged patterns^31^. This property combined with the ecological index aids to study the ecological characteristics of individual DCIS and is similarly used in histology studies to provide a spatial context of diverse cell types coexisting within the microenvironment^32^.

Let *K* be a set containing all coordinates of DCIS *D* and let (*D*_*k*_)_*k*_ ∈ _*K*_ be the coordinates of a DCIS*k*. A Voronoi region *R*_*k*_ generated by DCIS duct *D*_*k*_ contains all cells *P* that are not seeds and are closer to *D*_*k*_ than to any other seed *D*_*j*_, *j* ≠ *k*. Let *d*(*Q*_*i*_, *Q*_*j*_) be the Euclidean distance function between two centroids of DCIS *Q*_*i*_ and *Q*_*j*_ then

2 $$R_k = \{ x \in P\left| d \right.\left( {x,C_k} \right) \le d\left( {x,C_j} \right)\forall j\, \ne \,k\}.$$

Centroids from IM-Net segmentation were estimated and the resulting binary masks were mapped back to lower resolution (×1.25) of the image. The centroids were then used as a seed for calculating the Voronoi polygon. Because of these mathematical principles underlying Voronoi tessellation, lymphocytes within a polygon will be closer to its seed than any other seeds. This means that the closest DCIS duct for a lymphocyte is the one that ‘seeds’ the polygon containing this lymphocyte. Subsequently, lymphocytes and stromal cells within in situ microenvironments were reclassified as in situ lymphocytes and in situ stromal cells.

The Morisita–Horn similarity index is an ecological measure of community structure to quantify the extent of spatial colocalisation or overlap between two spatial variables. We have previously demonstrated its use in studying cancer-immune cell colocalisation in IDCs^19^. Here to measure colocalisation of DCIS and lymphocytes, the Morisita index was modified by restricting calculation in Voronoi polygons and estimating the density of epithelial cells and immune cells in the newly defined space. This space was further divided into Voronoi grids, following^19^. This is to provide more spatial points for calculation and to prevent a lack of power for samples with a low number of DCIS ducts. The number of immune cells and epithelial cells for each polygon *i* are denoted as $n_i^l$ and $n_i^e$, based on Voronoi tessellation. Let $p_i^l$ and $p_i^e$ denote the fraction of immune cells and epithelial cells in polygon *i*, i.e. $p_i^l = \frac{{n_i^l}}{{\mathop {\sum}\nolimits_i {n_i^l} }},\,p_i^e = \frac{{n_i^e}}{{\mathop {\sum}\nolimits_i {n_i^e} }}$. Morisita–Horn similarity index is henceforth referred to as DCIS immune colocalisation score throughout the manuscript and it is calculated as:

3 $${\mathrm{DCIS}}\,{\mathrm{immune}}\,{\mathrm{colocalisation}}\,{\mathrm{score}} = \frac{{2\mathop {\sum }\limits_i p_i^lp_i^e}}{{\mathop {\sum }\limits_i (p_i^l)^2 + \mathop {\sum }\limits_i (p_i^e)^2}}.$$

High DCIS immune colocalisation score indicates that TILs colocalise well with DCIS ducts within a sample, that is, spatial homogeneity; whereas, low DCIS immune colocalisation score could indicate TILs to only localise with part of the DCIS, i.e. high spatial variability.

### State-of-the-art networks for comparison

Single-shot detector^14^, Resnet 101-based RCNN network^15^, UNet^16^ and MicroNet^17^ were trained following optimal configuration as described in the original publications. Hyperparameters such as learning rate varied from 0.00001 to 0.001 based on both original publications and experiments. Further details on the number of parameters, optimiser and learning rate for an individual network of choice are provided in Supplementary Table 7.

### Quantitative evaluation at the slide level

We used DICE coefficient for our quantitative evaluation on the test dataset with the ground truth annotation. DICE measures the spatial overlap between the ground truth annotation and automated segmentation methods^33^. Quantitative measures include DICE, positive predictive value (PPV), negative predictive value (NPV), true positive rate (TPR), true negative rate (TNR), false-positive rate (FPR) and false-negative rate (FNR) for each test dataset across all slides.

### Biological validation

CK5 is an immunohistochemistry myoepithelial marker and can be used to differentiate between DCIS and invasive cancers^34^. To further evaluate the DCIS segmentation accuracy on H&E images, we generated eight pairs of H&E and CK5 immunohistochemistry (Dako, catalogue number M7237, lot number 20014544) slides from whole-tumour serial sections. Based on morphology and localisation of CK5 expression, areas of DCIS were hand-annotated, providing both quantity and area of individual DCIS for a quantitative evaluation for the automated segmentation results on H&E images of serial sections.

### Immune subset analysis

To gain additional knowledge on the immunological subtype of the lymphocytes present in these samples, we used serial sections of the IHC dataset stained for CD8, CD4 and FOXP3, respectively. CD4 and CD8 expression were defined by membranous lymphocyte labelling, and FOXP3 expression was defined by nuclear labelling of lymphocytes. DCIS immune colocalisation score was computed by restricting calculation in Voronoi polygons and estimating the density of epithelial cells and positive immune cell phenotype in the newly defined space. In addition, immune-epithelial cell colocalisation was computed following methods proposed in ref. ^19^ within the invasive cancer regions and in situ regions.

### Statistical methods

Wilcoxon test was used to determine the statistical significance of differences in immune scores between pure and adjacent DCIS samples, and *p*-value < 0.05 was used to determine significance. Patient-level statistical tests were also carried out using the Wilcoxon test by taking single WSI belonging to each group and randomised over 100 times to test for significance of the DCIS immune colocalisation score. Effect size to gauge the magnitude of experimental power was measured using Cohen’s *d*, for which *d* ≤ 0.2 is considered small, (0.2 < *d* ≤ 0.5) medium and *d* > 0.5 large^35,36^. Correlation analysis of parameters such as the estimated number of DCIS regions and the estimated area of DCIS was determined between automated H&E method and hand annotations based on CK5 IHC marker. Furthermore, leave one out cross-validation on the independent test set were performed to compute *R*-square average estimate and mean squared error for DCIS regions with the hand annotations from the IHC marker. All correlation tests used Spearman’s correlation method and statistical tests were carried out in R 3.6.1 and the cross-validation using python 3.6 for the models built using Tensorflow 1.8 and Keras 2.2.4.

### Reporting summary

Further information on research design is available in the Nature Research Reporting Summary linked to this article.

## Supplementary information

Supplementary Tables and Figures

Reporting Summary Checklist

## Data Availability

The data generated and analysed during this study are described in the following data record: 10.6084/m9.figshare.13007954^37^. All training data, including the fully anonymised raw H&E image tiles and pathological annotations as binary marks, as well as Python code, are available in the corresponding author’s GitHub: https://github.com/pathdata/UNMaSk. Requests for data access for the Duke samples can be submitted to E. Shelley Hwang (shelley.hwang@duke.edu) and Yinyin Yuan (yinyin.yuan@icr.ac.uk). Data underlying Figs. 4 and 6 are in the files ‘Ext_validData_DCIS_DAVE_Fig4_data.csv’ and ‘Ext_validData_DCIS_DAVE_Fig6_data.csv’, included with the figshare data record^37^. The images used as representative examples in Fig. 8 are listed in the file ‘Fig. 8 image details.xlsx’, included with the figshare data record^37^.

## Code availability

Python implementation of the UNMaSk pipeline is maintained in GitHub repository: https://github.com/pathdata/UNMaSk for reproducibility.
