# Supplementary material for: Unmasking the immune microecology of ductal carcinoma in situ with deep learning
Source: NPJ Breast Cancer. 2021 Mar 1;7:19. doi: 10.1038/s41523-020-00205-5 (PMC7921670; doi:10.1038/s41523-020-00205-5)
Supplement: Supplementary file 1 — Supplementary Tables and Figures [file 41523_2020_205_MOESM1_ESM.pdf]

Unmasking the immune microecology of ductal carcinoma *in situ* with deep learning

Priya Lakshmi Narayanan[1,2]*, Shan E Ahmed Raza[1,2], Allison H. Hall[3], Jeffrey R. Marks[4], Lorraine King[4], Robert B. West[5], Lucia Hernandez[6], Naomi Guppy[7,8], Mitch Dowsett[9,10], Barry Gusterson[1], Carlo Maley[11], E. Shelley Hwang[4], Yinyin Yuan[1, 2]*

*: Joint corresponding authors

[1] Centre for Evolution and Cancer, Institute of Cancer Research, UK

[2] Division of Molecular Pathology, Institute of Cancer Research, UK

[3] Department of Pathology, Duke University School of Medicine, Durham, North Carolina, USA

[4] Department of Surgery, Duke University School of Medicine, Durham, North Carolina, USA

[5] Department of Pathology, Surgical Pathology, Stanford, California, USA

[6] Hospital Universitario, 12 de Octubre, Department of Anatomic Pathology, Madrid, Spain

[7] Breast Cancer Now Histopathology Core, Institute of Cancer Research, UK

[8] UCL Advanced Diagnostics, University College London, UK

[9] The Breast Cancer Now Toby Robins Research Centre, Institute of Cancer Research,

[10] Academic Department of Biochemistry, Royal Marsden Hospital, UK

[11] Biodesign Center for Personalized Diagnostics and School of Life Sciences, Arizona State University, Tempe, Arizona, USA

Supplementary Table 1. Breakdown of performance of tissue segmentation indicating segmentation accuracy based on Dice coefficient.

Model	TPR	TNR	FNR	FPR	PPV	NPV	Dice
Threshold	0.72±0.10	0.50±0.10	0.27±0.10	0.49±0.10	72.4±10.2	50.7±10.8	0.73±0.15
**UNet**	0.72±0.10	0.76±0.06	0.28±0.10	**0.23±0.06**	**71.8±10.5**	**76.5±6.06**	**0.78±0.10**

Supplementary Table 2: Quantitative comparison with state-of-the-art methods and the classification accuracy of deep learning networks for DCIS detection on test dataset split at patient level.

Model	Precision	Recall	F1-score
SSD	0.79	0.63	0.70
Faster RCNN with Resnet	0.81	0.65	0.72
UNet	0.79	0.64	0.71
MicroNet	0.82	0.72	0.77
IM-Net	0.83	0.75	0.79

Supplementary Table 3. Evaluation using three fold cross validation stratified at patient level in each fold of training images from both TransATAC and Duke dataset.

IM-Net	Precision	Recall	F1-score
Fold 1	0.854	0.781	0.815
Fold 2	0.861	0.746	0.795
Fold 3	0.866	0.765	0.811
**MicroNet**	**Precision**	**Recall**	**F1-score**
Fold 1	0.781	0.758	0.769
Fold 2	0.842	0.702	0.765
Fold 3	0.846	0.714	0.774

Supplementary Table 4: Quantitative comparison of performance metric of TransATAC dataset used as training and Duke dataset as testing set.

Model	Precision	Recall	F1-score
MicroNet	0.73	0.78	0.754
IM-Net	0.79	0.81	0.8

Supplementary Table 5. Breakdown of detection and segmentation performance of convolution networks on 18 whole slide images indicating accuracy.

**Model**	**TPR**	**TNR**	**FNR**	**FPR**	**PPV**	**NPV**	**Dice**
SSD	0.70 ± 0.17	0.95 ± 0.09	0.24 ± 0.05	0.40 ± 0.15	69.29 ± 0.2	94.59 ± 0.2	0.65 ± 0.7
Faster RCNN with ResNet	0.71 ± 0.16	0.98 ± 0.04	0.27 ± 0.06	0.13 ± 0.02	71.2 ± 0.15	97.75 ± 0.03	0.71 ± 0.65
UNet	0.73 ± 0.18	0.97 ± 0.08	0.28 ± 0.05	0.08 ± 0.10	72.8 ± 0.20	98.2 ± 0.07	0.77 ± 0.30
MicroNet	0.75 ± 0.12	0.98 ± 0.02	0.24 ± 0.03	0.013 ± 0.01	75.79 ± 0.3	98.60 ± 0.03	0.82 ± 0.50
**IM-Net**	**0.77 ± 0.10**	**0.98 ± 0.05**	**0.24 ± 0.01**	**0.011 ± 0.02**	**76.8 ± 0.1**	**98.50 ± 0.01**	**0.83 ± 0.30**

Supplementary Table 6. Evaluation of five methods for DCIS segmentation using correlation metrics (number of DCIS detected and areas segmented versus expert annotations), and Mean Squared Error (MSE) and R-square estimate of segmented area to assess boundary detection accuracy.

	**IM-Net**	MicroNet	UNet	RFCNN	SSD
Number of DCIS	**0.97**	0.97	0.95	0.93	0.92
Segmented Area	**0.99**	0.99	0.98	0.96	0.94
MSE of segmented area	**0.17**	0.25	0.29	0.34	0.41
R-Square estimate	**0.65**	0.59	0.55	0.38	0.31

Supplementary Table 7. Details of patch size and hyper-parameters for DCIS detection and segmentation.

**Model**	**Input patch size**	**Total number of parameters**	**Learning rate**	**Optimizer**
**SSD**	224x224	27,304,700	0.0001	RMSprop optimizer
**RCNN**	224x224	68,607,459	0.0003	Momentum optimizer
**UNet**	252x252	15,394,825	0.001	Adagrad optimizer
**MicroNet**	508x508	912,514,125	0.001	Adagrad optimizer
**IM-Net**	508x508	913,415,373	0.0001	Adagrad optimizer

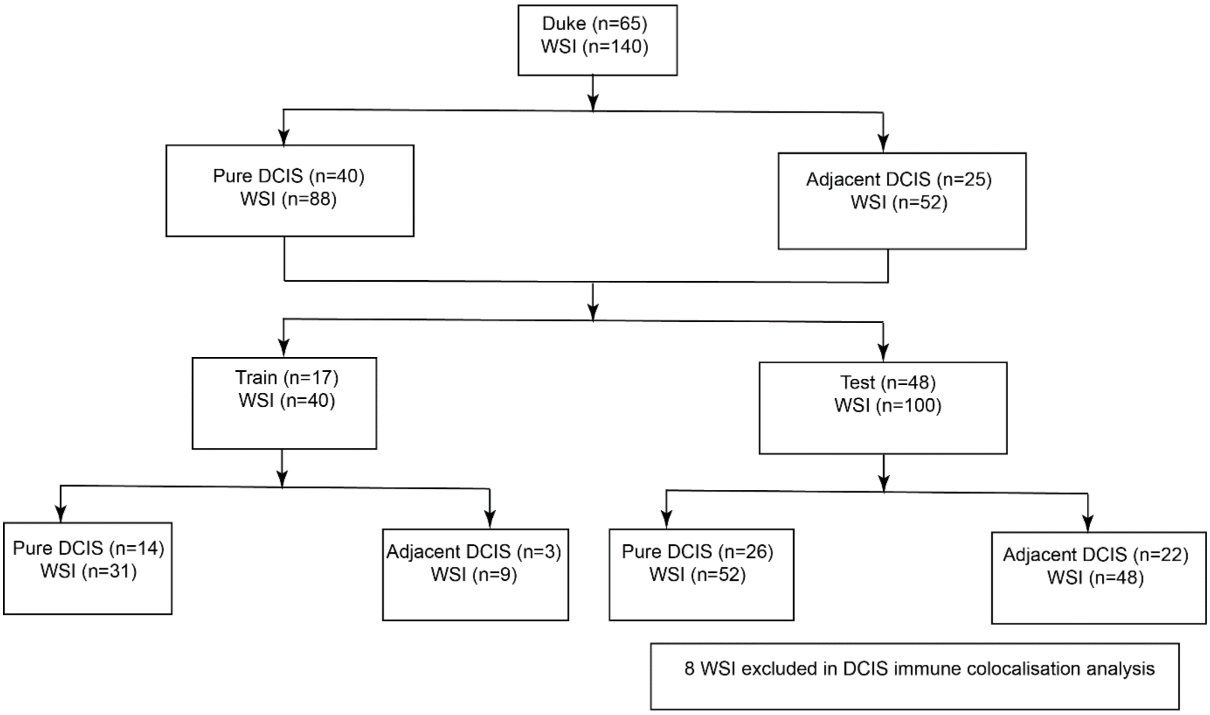

Supplementary Figure 1. Flow schema of the Duke dataset.

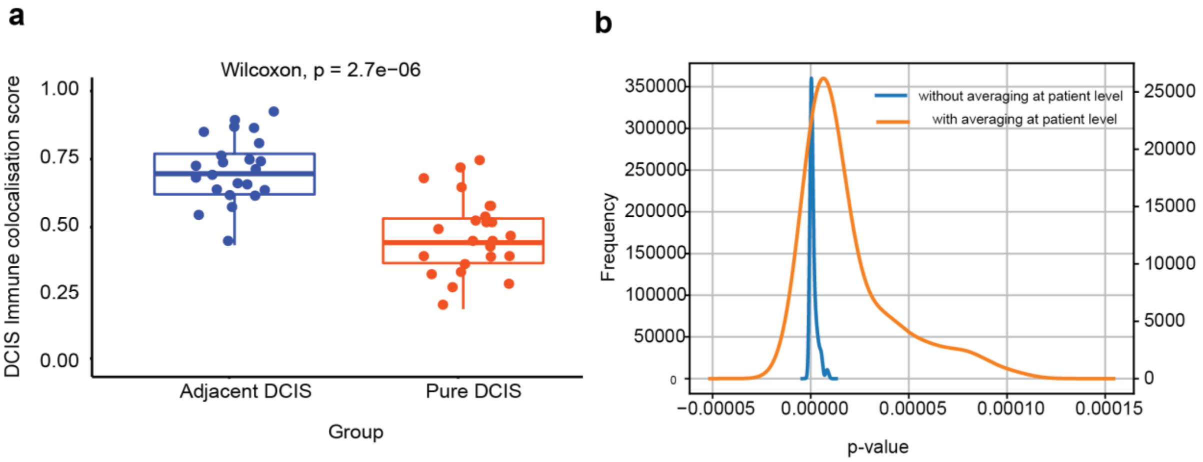

Supplementary Figure 2. Comparison of TIL distribution pattern local to DCIS ducts in adjacent versus pure DCIS cases after averaging per patient or by means of random sampling per patient. (a) Patient level Morisita score was averaged from one/two/three images per patient belonging to 40 patients of pure DCIS and 25 patients of adjacent DCIS in total. (b) Distribution of p-values in 100 random sampling for calculated patient level scores.

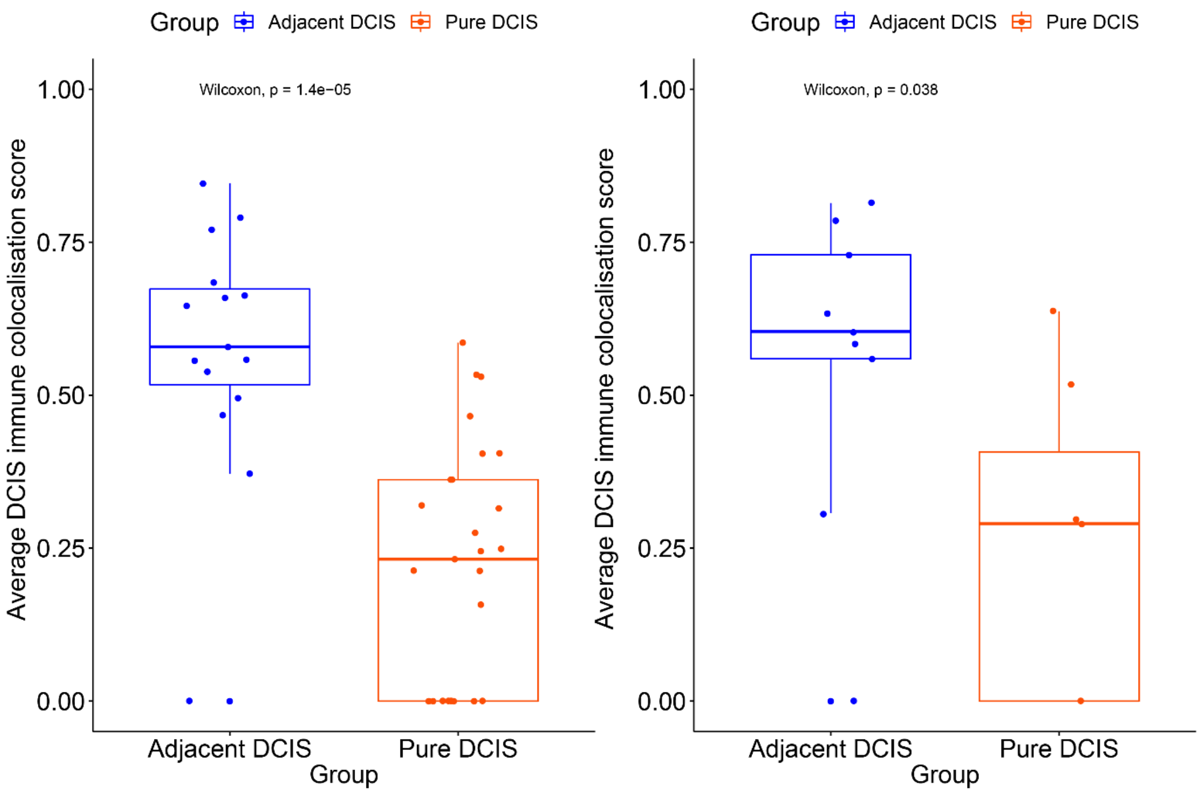

Supplementary Figure 3. Boxplots illustrating the difference in DCIS-immune colocalisation score at patient level between pure DCIS and adjacent DCIS for ER+ (left) and ER- (right) tumours.

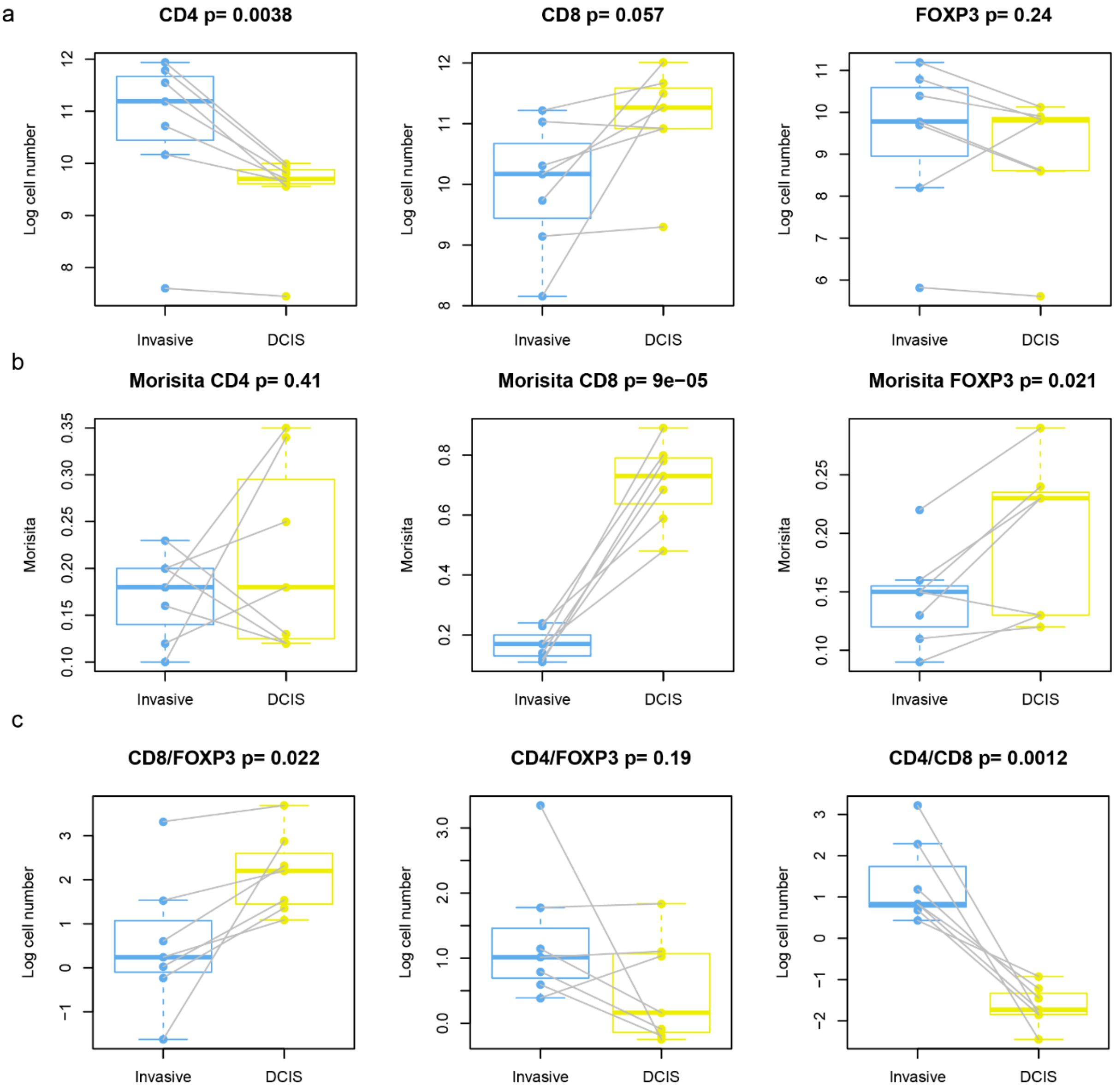

Supplementary Figure 4. Box and whisker plots indicating TIL phenotypes observed in Invasive and DCIS region of adjacent DCIS dataset. (a) Immune cell proportion of CD4, CD8 and FOXP3 demonstrating immune cell type differences in invasive and DCIS region using paired tests. (b) DCIS immune colocalisation score differences observed using paired test between the Invasive and DCIS region. (c) Ratio of immune cell types demonstrating differences in invasive and DCIS region using paired tests.

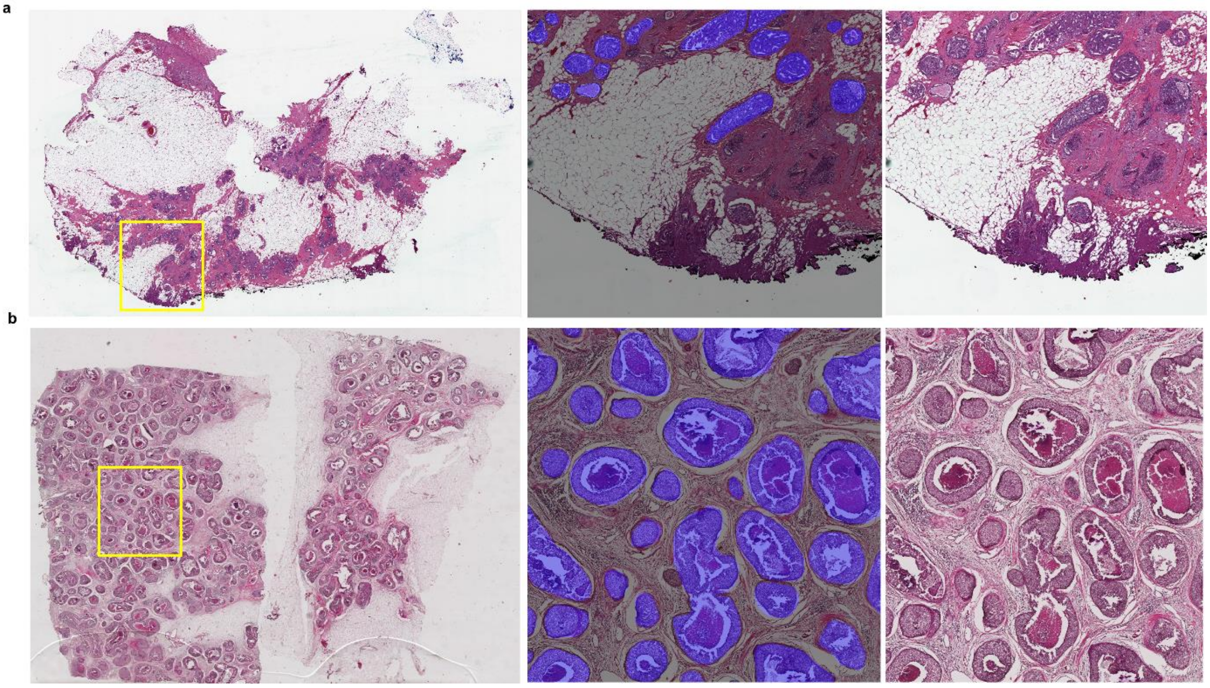

Supplementary Figure 5. Illustrative examples from the test dataset comprising (a) solid and cribiform DCIS without necrosis and (b) DCIS regions with necrosis, where Dice score of (a) 0.91 and (b) 0.87 was achieved by IM-Net. Left: original image at a 1.25x magnification. Middle: High power field of IM-Net segmentation comprising DCIS region of area 4mm^2. Right: High power field of tiled image of area 4mm^2.

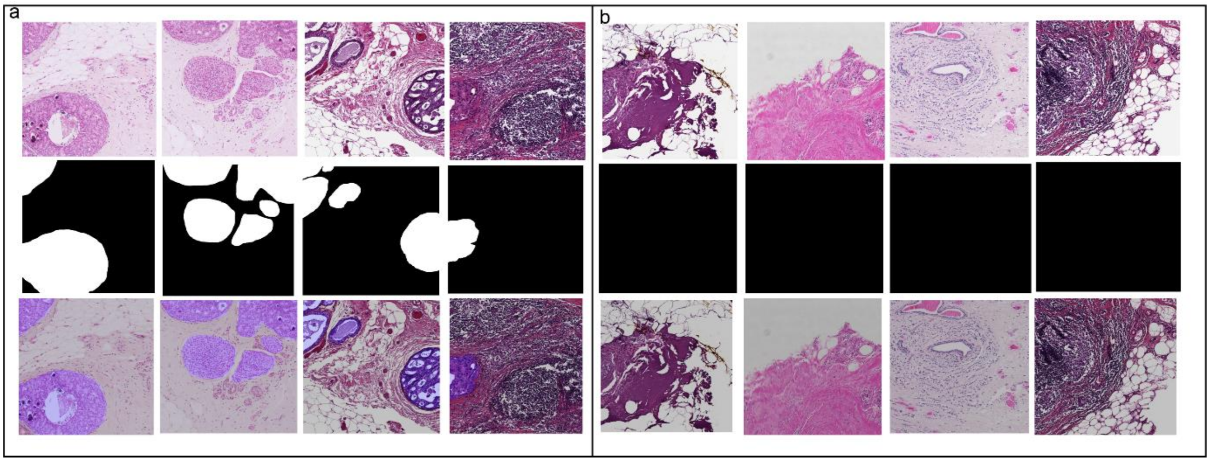

Supplementary Figure 6. Representative images of positive and negative examples with heterogeneous microenvironment. DCIS regions were marked as foreground (violet); representative heterogeneous negative images with only background pixels set to zero. (a) Left top row: representative positive examples; Left middle row: positive regional mask; Left bottom row: positive regional mask overlay image highlighting the foreground pixels in violet for visualisation with transparency of 0.6. (b) Right top row: representative negative images; Right middle row: regional mask with all pixels set to zero; Right bottom row: regional mask as overlay image with transparency of 0.6 for visualisation.
