# Supplementary material for: Unmasking the immune microecology of ductal carcinoma in situ with deep learning
Source: NPJ Breast Cancer. 2021 Mar 1;7:19. doi: 10.1038/s41523-020-00205-5 (PMC7921670; doi:10.1038/s41523-020-00205-5)
Supplement: Supplementary file 2 — Reporting Summary Checklist [file 41523_2020_205_MOESM2_ESM.pdf]

nature research

Corresponding author(s): NPJBCANCER-00587R; Priya Lakshmi Narayanan and Yinyin Yuan

Last updated by author(s): Jun 16, 2020

Reporting Summary

Nature Research wishes to improve the reproducibility of the work that we publish. This form provides structure for consistency and transparency in reporting. For further information on Nature Research policies, see our Editorial Policies and the Editorial Policy Checklist.

Statistics

For all statistical analyses, confirm that the following items are present in the figure legend, table legend, main text, or Methods section.

n/a	Confirmed	
☐	☒	The exact sample size (n) for each experimental group/condition, given as a discrete number and unit of measurement
☐	☒	A statement on whether measurements were taken from distinct samples or whether the same sample was measured repeatedly
☐	☒	The statistical test(s) used AND whether they are one- or two-sided<br>*Only common tests should be described solely by name; describe more complex techniques in the Methods section.*
☒	☐	A description of all covariates tested
☒	☐	A description of any assumptions or corrections, such as tests of normality and adjustment for multiple comparisons
☒	☐	A full description of the statistical parameters including central tendency (e.g. means) or other basic estimates (e.g. regression coefficient) AND variation (e.g. standard deviation) or associated estimates of uncertainty (e.g. confidence intervals)
☐	☒	For null hypothesis testing, the test statistic (e.g. F, t, r) with confidence intervals, effect sizes, degrees of freedom and P value noted<br>*Give P values as exact values whenever suitable.*
☒	☐	For Bayesian analysis, information on the choice of priors and Markov chain Monte Carlo settings
☒	☐	For hierarchical and complex designs, identification of the appropriate level for tests and full reporting of outcomes
☐	☒	Estimates of effect sizes (e.g. Cohen's d, Pearson's r), indicating how they were calculated

Our web collection on statistics for biologists contains articles on many of the points above.

Software and code

Policy information about availability of computer code

Data collection	Main source of data collection is microscopic HE and IHC slide scanned at 20X magnification. NanoZoomer (Hamamatsu, Japan) was used for digitalizing HE and IHC slides. Aperio was used for digitalizing DUKE HE slides.
Data analysis	The deep learning pipeline for digital pathology image analysis is available for non-commercial research purposes: https://github.com/pathdata/HE_Tissue_Segmentation. The pipeline runs on Python (version 3.5), TensorFlow (version 1.8) and MATLAB (version R2018b).

For manuscripts utilizing custom algorithms or software that are central to the research but not yet described in published literature, software must be made available to editors and reviewers. We strongly encourage code deposition in a community repository (e.g. GitHub). See the Nature Research guidelines for submitting code & software for further information.

Data

Policy information about availability of data

All manuscripts must include a data availability statement. This statement should provide the following information, where applicable:

- Accession codes, unique identifiers, or web links for publicly available datasets
- A list of figures that have associated raw data
- A description of any restrictions on data availability

Digital pathology images from Duke study can be obtained upon reasonable request sent by email to Y.Y and E.S.H. A test subset of such digital pathology images at a smaller resolution are uploaded to github branch https://github.com/pathdata/HE_Tissue_Segmentation.

Field-specific reporting

Please select the one below that is the best fit for your research. If you are not sure, read the appropriate sections before making your selection.

☒ Life sciences ☐ Behavioural & social sciences ☐ Ecological, evolutionary & environmental sciences

For a reference copy of the document with all sections, see nature.com/documents/nr-reporting-summary-flat.pdf

Life sciences study design

All studies must disclose on these points even when the disclosure is negative.

Sample size	No statistical methods were used to predetermine sample size. For DUKE, TransATAC and IHC datasets, all patient tumor regions and ductal carcinoma in situ regions with histology of sufficient quality were included in this study.
Data exclusions	1 whole slide from IHC dataset was excluded due to poor quality of the image.
Replication	Deep learning analysis on a smaller subset of test data, cross validated by selecting the best model and then scaled up on remaining test slides of DUKE and IHC dataset.
Randomization	Not relevant to the study.
Blinding	Not relevant to the study.

Reporting for specific materials, systems and methods

We require information from authors about some types of materials, experimental systems and methods used in many studies. Here, indicate whether each material, system or method listed is relevant to your study. If you are not sure if a list item applies to your research, read the appropriate section before selecting a response.

Materials & experimental systems

n/a	Involved in the study	
☐	☒	Antibodies
☒	☐	Eukaryotic cell lines
☐	☐	Palaeontology and archaeology
☒	☐	Animals and other organisms
☐	☒	Human research participants
☒	☐	Clinical data
☒	☐	Dual use research of concern

Methods

n/a	Involved in the study	
☒	☐	ChIP-seq
☒	☐	Flow cytometry
☒	☐	MRI-based neuroimaging

Antibodies

Antibodies used	For T cell IHC slides (CD8/CD4/FOXP3) were used: CD8 (clone 4B11, Leica Ready To Use PA0183); CD4 (clone 4B12, Leica Ready To Use PA0427); FOXP3 (clone PCH101, eBioscience 14-4776-82); CK5 Dako, catalogue number M7237, lot number 20014544 were used.
Validation	CD8: This was verified in-house on a series of 20 lymphoid neoplasms (10 expected positive, 10 expected negative), normal tonsil, normal lymph node and non-lymphoid organs. This is a CE-IVD antibody used in line with manufacturer's instructions, and was assessed by participation in UKNEQAS ICC&ISH Lymphoma module, with no poor results.<br>CD4: This was verified similarly to CD8 above;This is a CE-IVD antibody used in line with manufacturer's instructions and was assessed by UKNEQAS as per CD8 above.<br>FOXP3: This was verified on a series of lymphoma cases.<br>CK5: This was verified in-house on 5 samples each of human prostate, tonsil and breast, 5 cases of breast cancer and 5 murine mammary fat pads.

Palaeontology and Archaeology

Specimen provenance	Not relevant
Specimen deposition	Not relevant

Dating methods | Not relevant

☐ Tick this box to confirm that the raw and calibrated dates are available in the paper or in Supplementary Information.

Ethics oversight | Not relevant

Note that full information on the approval of the study protocol must also be provided in the manuscript.

Human research participants

Policy information about studies involving human research participants

Population characteristics | 65 patients were part of Duke study. Flow diagram is shown in supplementary Figure S1 and Patient demographics are summarized in Table 1 of the manuscript.

Recruitment | *Describe how participants were recruited. Outline any potential self-selection bias or other biases that may be present and how these are likely to impact results.*

Ethics oversight | *Identify the organization(s) that approved the study protocol.*

Note that full information on the approval of the study protocol must also be provided in the manuscript.
